# Polygonumnolides A1–B3, minor dianthrone derivatives from the roots of *Polygonum multiflorum* Thunb

**DOI:** 10.1007/s12272-016-0816-7

**Published:** 2017-07-05

**Authors:** Jianbo Yang, Zheng Yan, Jin Ren, Zhong Dai, Shuangcheng Ma, Aiguo Wang, Yalun Su

**Affiliations:** 1 State Key Laboratory of Bioactive Substance and Function of Natural Medicines, Institute of Materia Medica, Chinese Academy of Medical Sciences and Peking Union Medical College, Beijing, 100050 People’s Republic of China; 2 Research and Inspection Center of Traditional Chinese Medicine and Ethnomedicine, National Institutes for Food and Drug Control, State Food and Drug Administration, Beijing, 100050 People’s Republic of China; 3 School of Chinese Pharmacy, Beijing University of Chinese Medicine, Beijing, 100102 People’s Republic of China

**Keywords:** *Polygonum multiflorum* Thunb., Dianthrone glycosides, Polygonumnolides A1–B3, KB tumor cell lines

## Abstract

Seven new dianthrone glycosides, named polygonumnolides A1–B3 (**1**–**7**), were isolated from the 70 % EtOH extract of the dried roots of *Polygonum multiflorum* Thunb. using column chromatography and preparative high-performance liquid chromatography. Their structures were determined by 1D and 2D NMR and mass spectroscopy. The isolated compounds were evaluated for their cytotoxic effects against KB tumor cell lines and compounds **1**–**4** showed moderate cytotoxicity.

**Electronic supplementary material:**

The online version of this article (doi:10.1007/s12272-016-0816-7) contains supplementary material, which is available to authorized users.

## Introduction

The roots of *Polygonum multiflorum* Thunb. are used as a traditional Chinese medicinal herb to treat many diseases. They are applied as remedies for preventing hair loss and premature graying, strengthening bones and muscles, and treating seminal emission and menstrual and menopausal complaints (The State Pharmacopoeia Commission 2015). Chemical extractions of the roots of *P. multiflorum* resulted in the isolation of approximately 100 compounds (Lin et al. 2015), including anthraquinones, stilbenes, phenolic acid, phospholipids and flavones. In our continuing search for bioactive compounds, 70 % Et OH extract of the dried roots of *P. multiflorum* was investigated. Seven new dianthrone glycosides, named polygonumnolides A1–B3 (**1**–**7**), were isolated, and the structural elucidation of these new compounds are described herein as well as their cytotoxic effects against KB tumor cell lines.

## Materials and methods

### General experimental procedures

Optical rotations were acquired on a Jasco P-2000 polarimeter (Jasco Inc., Tokyo, Japan). UV data were recorded using a Jasco V-650 spectrophotometer (Jasco Inc., Tokyo, Japan). Experimental electronic circular dichroism spectra (ECD) were recorded on a Chirascan spectrophotometer. IR spectra were measured on a Nicolet iN 10 Micro FTIR spectrophotometer (Thermo Nicolet Inc., Waltham, MA, USA). NMR spectra were recorded on Varian Inova-300, 500 and 600 spectrophotometers (Varian Inc., Palo Alto, CA, USA). HRESI-MS were obtained using an Agilent 1100 UPLC-Q-TOF mass spectrometer (Agilent Technologies Ltd., Santa Clara, CA, USA). Column chromatography was performed with silica gel (200–300 mesh; Qingdao Marine Chemistry Company, Qingdao, China), Sephadex LH-20 (GE Healthcare Bio-Sciences AB, Uppsala, Sweden) and reversed-phase C_18_ silica gel (40–60 µm, Alltech, Deerfield, IL, USA). Preparative high-performance liquid chromatography (HPLC) separations were carried out using a Shimadzu LC-10 A system equipped with a YMC-Pack ODS-A column (250 × 20 mm, 5 µm, Kyoto, Japan) and a Shimadzu SPD-20 A detector (Shimadzu).

### Plant material

The dried roots of *P. multiflorum* Thunb. were collected from Deqing, Guangdong Province, People’s Republic of China, in October 2012, and identified by associate Prof. Ji Zhang (Research and Inspection Center of Traditional Chinese Medicine and Ethnomedicine, National Institutes for Food and Drug Control, State Food and Drug Administration). A voucher specimen (No. 060104) has been deposited at the Research and Inspection Center of Traditional Chinese Medicine and Ethnomedicine, National Institutes for Food and Drug Control, State Food and Drug Administration, Beijing 100050, People’s Republic of China.

### Extraction and isolation

The roots of *P. multiflorum* Thunb. (28.0 kg) were extracted three times with 70 % EtOH under reflux and filtered. The filtrate was evaporated under reduced pressure at 50 °C to afford a crude extract (4.0 kg). The crude extract was partitioned with CH_2_Cl_2_ and H_2_O. The H_2_O fraction (3.5 kg) was loaded onto macroporous resin (DM-8) and eluted with a gradient of water and 95 % EtOH mixture (H_2_O, 25 % EtOH, 40 % EtOH, 55 % EtOH and 95 % EtOH) to give five fractions (A–E; fraction A: 2.0 kg; fraction B: 62.0 g; fraction C: 200.0 g; fraction D: 38.0 g; fraction E: 55.0 g). Fraction E was separated on a Sephadex LH-20 column using step gradient elution of methanol–water (from 60 to 100 % v/v) to give five fractions (A_1_–A_5_) based on RP-TLC analysis. Fraction A_3_ was further chromatographed on a RP-18 silica gel column using a step gradient elution of methanol–water (from 40 to 100 % v/v) to give 5 fractions (B_1_–B_5_) based on RP-TLC analysis.

Fraction B_4_ was purified on a Sephadex LH-20 column (100 % MeOH) to give fractions C_4_–C_5_ and isolated 2* (6.5 mg) and 4* (6.8 mg). Fraction C_4_ (50 mg) was further separated and purified by preparative HPLC (CH_3_CN/H_2_O, 50:50; YMC, 250 × 20 nm, S-5 µm; 210 nm; 5.0 mL/min) to yield 3* (7.0 mg, 54.0 min). Fraction C_5_ (50.0 mg) was separated and purified by preparative HPLC (CH_3_CN/H_2_O, 60:40; YMC, 250 × 20 nm, S-5 µm; 210 nm; 5.0 mL/min) to yield 1* (7.0 mg, 28.0 min). Fraction B_2_ was separated and purified by preparative HPLC (CH_3_CN/H_2_O, 35:65; YMC, 250 × 20 nm, S-5 µm; 210 nm; 5.0 mL/min) to yield 5* (7.0 mg, 31.0 min), 6* (6.0 mg, 36.0 min) and 7* (6.0 mg, 60.0 min), respectively.

### Polygonumnolide A1 (**1**)

Yellow powder; [α]_D_^25^ −152° (c = 0.1, MeOH); UV (MeOH) λ_max_ (logε): 207 (4.87), 280 (4.27), 351 (4.31) nm; ECD (c = 1.61 × 10^−4^ M, MeOH): Δ*ε*_392.0 nm_ −15.55, Δ*ε*_338.0 nm_ +20.35, Δ*ε*_279.0 nm_ −16.75; IR (KBr) ν_max_: 3361, 2919, 1620, 1599, 1489, 1376, 1333, 1254, 1176, 1160, 1074, 908, 862, 788 cm^−1^; ^1^H NMR (CD_3_OD, 500 MHz) and ^13^C NMR (CD_3_OD, 125 MHz) data, see Tables 1 and 2; HRESI–MS: *m/z* 685.1940 [M–H]^−^ (calcd for C_37_H_34_O_13,_ 685.1921).

**Table 1:** ^1^HNMR spectroscopic data of compounds **1**–**4**

No	1*^a^	2*^b^	3*^c^	4*^b^
2	6.45, s	6.60, s	6.56, s	6.51, s
4	5.58, br s	6.16, br s	5.89, br s	5.85, br s
5	6.30, br s	6.38, br s	6.70, br s	6.56, br s
7	6.83, d (2.0)	7.02, d (1.8)	7.02, d (2.0)	7.00, d (2.4)
10	4.14, d (3.5)	4.57, d (3.6)	4.55, s	4.39, d (3.0)
3-Me	2.07, s	2.24, s	2.17, s	2.17, s
2′	6.59, s	6.71, s	6.57, s	6.53, s
4′	6.34, br s	6.38, br s	5.98, br s	5.91, br s
5′	5.45, br s	6.15, br s	6.55, br s	6.18, br s
7′	6.18, d (2.5)	6.31, d (1.8)	6.43, d (2.5)	6.18, s
10′	4.08, d (3.5)	4.56, d (3.6)	4.55, s	4.35, d (3.0)
3′-Me	2.29, s	2.31, s	2.22, s	2.17, s
1″	4.60, d (7.5)	4.70, d (7.2)	4.75, d (8.0)	4.76, d (7.2)
2″	3.43, m	3.49, m	3.47, m	3.51,m
3″	3.43, m	3.50, m	3.59, m	3.51, m
4″	3.38, m	3.46, m	3.59, m	3.38, m
5″	3.49, m	3.47, m	3.47, m	3.50, m
6″	3.94, dd (2.5,12) 3.74, dd (5.5,12)	3.96, dd (1.2,11.4) 3.77, dd (5.4,11.4)	3.98, dd (2.5,13) 3.77, dd (5.5,13)	3.94, dd (2.4, 12); 3.71, dd (6.0,12)
6′-0Me	3.74, s	3.84, s	3.95, s	3.88, s
OH-1		12.68, s	12.02, s	
OH-8′		11.96, s	12.04, s	
OH-1′		11.82, s	11.66, s	
OH-6		9.89, s		
OH-6′				

^a^ ^1^HNMR data were measured in CD_3_OD at 500 MHz

^b^ ^1^HNMR data were measured in CD_3_COCD_3_ or CD_3_OD at 600 MHz

^c^ ^1^HNMR data were measured in CD_3_COCD_3_ at 500 MHz

**Table 2:** ^13^C NMR spectroscopic data of compounds **1**–**4**

No	1*^a^	2*^b^	3*^c^	4*^b^
1	162.3	163.1	162.2	162.2
2	117.3	117.1	117.1	117.6
3	146.8	146.4	146.3	147.1
4	121.8	121.1	121.4	121.8
5	112.5	112.2	112.0	110.9
6	164.6	163.6	163.9	165.7
7	107.7	106.6	107.2	105.9
8	161.9	161.8	161.9	161.6
9	188.5	188.2	188.2	188.6
10	57.7	57.2	57.2	58.0
1a	117.5	116.8	117.4	118.1
4a	143.0	142.2	140.7	141.1
5a	146.4	145.9	146.1	146.5
8a	117.1	116.3	116.7	117.6
3-Me	21.8	21.8	21.7	21.9
1′	162.6	162.6	162.8	162.8
2′	117.7	117.3	117.3	117.4
3′	148.3	147.8	147.7	147.5
4′	122.2	122.1	122.0	122.2
5′	109.7	108.3	108.9	110.7
6′	166.4	166.4	166.8	167.8
7′	100.7	101.5	101.1	103.3
8′	165.7	165.4	165.3	165.7
9′	191.3	191.4	191.2	191.0
10′	56.7	56.4	56.3	56.7
1a′	116.0	115.2	114.7	115.3
4a′	140.0	140.7	139.7	140.3
5a′	143.1	144.4	145.4	145.8
8a′	111.5	111.4	112.2	111.1
3′-Me	22.1	22.0	22.2	22.3
1″	106.1	105.1	106.5	105.9
2″	74.9	74.5	74.9	74.9
3″	78.8	78.4	78.6	78.9
4″	71.2	71.3	71.3	71.5
5″	77.4	77.1	77.1	77.4
6″	62.7	62.8	62.9	62.7
6′-0Me	56.5	56.2	56.2	56.4

^a^ ^13^CNMR data were measured in CD_3_OD at 125 MHz

^b^ ^13^CNMR data were measured in CD_3_COCD_3_ or CD_3_OD at 150 MHz

^c^ ^13^CNMR data were measured in CD_3_COCD_3_ at 125 MHz

### Polygonumnolide A2 (**2**)

Yellow powder; [α]_D_^25^ −27.3° (c = 0.11, MeOH); UV (MeOH) λ_max_ (logε): 207 (4.88), 279 (4.26), 351 (4.31) nm; ECD (c = 1.33 × 10^−4^ M, MeOH): Δ*ε*_387.0 nm_ +5.54, Δ*ε*_341.5 nm_ −5.12, Δ*ε*_317.0.0 nm_ +4.12, Δ*ε*_295.0 nm_ −5.76, Δ*ε*_278.0 nm_ +3.88; IR (KBr) ν_max_: 3404, 2924, 1619, 1599, 1488, 1378, 1334, 1256, 1218, 1177, 1074, 906, 861, 799 cm^−1^; ^1^H NMR (CD_3_COCD_3_, 600 MHz) and ^13^C NMR (CD_3_COCD_3_, 150 MHz) data, see Tables 1 and 2; HRESI-MS: *m/z* 685.1906 [M–H]^−^ (calcd for C_37_H_34_O_13,_ 685.1921).

### Polygonumnolide A3 (**3**)

Yellow powder; [α]_D_^25^ −220° (c = 0.10, MeOH); UV (MeOH) λ_max_ (logε): 207 (4.86), 279 (4.26), 351 (4.30) nm; ECD (c = 1.33 × 10^−4^ M, MeOH): Δ*ε*_366.5 nm_ +9.17, Δ*ε*_316.0.5 nm_ −14.47, Δ*ε*_288.0 nm_ +0.81, Δ*ε*_276.0 nm_ −2.28; IR (KBr) ν_max_: 3391, 2921, 1619, 1598, 1489, 1369, 1332, 1252, 1177, 1160, 1072, 1036, 907, 860, 788 cm^−1^; ^1^H NMR (CD_3_COCD_3_, 500 MHz) and ^13^C NMR (CD_3_COCD_3_, 125 MHz) data, see Tables 1 and 2; HRESI-MS: *m/z* 685.1943 [M–H]^−^ (calcd for C_37_H_34_O_13,_ 685.1921).

### Polygonumnolide A4 (**4**)

Yellow powder; [α]_D_^25^ −250° (c = 0.06, MeOH); UV (MeOH) λ_max_ (logε): 207 (4.86), 279 (4.25), 351 (4.29) nm; ECD (c = 1.33 × 10^−4^ M, MeOH): Δ*ε*_390.0 nm_ −14.66, Δ*ε*_343.0.5 nm_ +17.65, Δ*ε*_305.0 nm_ −17.56, Δ*ε*_280.0 nm_ +8.44, Δ*ε*_267.5 nm_ +7.77; IR (KBr) ν_max_: 3375, 2918, 1619, 1600, 1489, 1372, 1331, 1252, 1176, 1160, 1073, 1036, 907, 861, 789 cm^−1^; ^1^H NMR (CD_3_OD, 500 MHz) and ^13^C NMR (CD_3_OD, 125 MHz) data, see Tables 1 and 2; HRESI-MS: *m/z* 685.1898 [M–H]^−^ (calcd for C_37_H_34_O_13,_ 685.1921).

### Polygonumnolide B1 (**5**)

Yellow powder; [α]_D_^25^ −120° (c = 0.10, MeOH); UV (MeOH) λ_max_ (logε): 208(4.81), 279 (4.41), 348 (4.38) nm; ECD (c = 1.95 × 10^−4^ M, MeOH): Δ*ε*_382.0 nm_ −13.84, Δ*ε*_319.0 nm_ +28.56, Δ*ε*_279.5 nm_ −16.53; IR (KBr) ν_max_: 3381, 2922, 1630, 1599, 1493, 1354, 1334, 1259, 1217, 1178, 1075, 906, 865, 793 cm^−1^; ^1^H NMR (CD_3_OD, 600 MHz) and ^13^C NMR (CD_3_OD, 150 MHz) data, see Tables 3 and 4; HRESI-MS: *m/z* 847.2453 [M–H]^−^ (calcd for C_43_H_44_O_18,_ 847.2454).

**Table 3:** ^1^H NMR spectroscopic data of compounds **5**–**7**

No	5*^a^	6*^a^	7*^a^
2	6.52, s	6.65, s	6.60, s
4	5.63, br s	5.96, br s	6.49, br s
5	6.48, br s	6.19, br s	5.60, br s
7	6.85, s	6.97, d (2.4)	6.75, d (1.8)
10	4.30, s	4.39, d (3.0)	4.25, s
3-Me	2.12, s	2.30 s	2.33 s
2′	6.64, s	6.57, s	6.63, s
4′	6.54, br s	6.25, br s	6.49, br s
5′	5.78, br s	6.19, br s	5.71, br s
7′	6.88, d (1.2)	6.83, d (1.8)	6.88, d (1.8)
10′	4.32, s	4.32, d (3.0)	4.25, s
3′-Me	2.37, s	2.20, s	2.34, s
6′-OMe	3.78, s	3.81, s	3.72, s
1″	4.64, d (7.2)	4.67, d (7.8)	4.89, d (7.8)
2″	3.47, m	3.49, m	3.48, m
3″	3.45, m	3.46, m	3.60, m
4″	3.38, m	3.44, m	3.43, m
5″	3.44, m	3.55, m	3.67, m
6″	3.99, dd (1.8,10.8); 3.81, dd (5.4,10.8);	4.00, dd (1.8,12.6); 3.79,dd (5.4,12.6)	3.96, dd (1.8,12) 3.76, dd (5.4,12)
1′′′	4.67, d (7.8)	4.73, d (7.2)	4.94, d (7.2)
2′′′	3.58, m	3.59, m	3.50, m
3′′′	3.45, m	3.46, m	3.58, m
4′′′	3.44, m	3.44, m	3.39, m
5′′′	3.54, m	3.55, m	3.69, m
6′′′	3.95, dd (1.8,12); 3.73, dd (6.0,12)	3.97, dd (1.8,12); 3.77,dd (6.0, 12)	3.96, dd (1.8,12); 3.72,dd (6.0, 12)

^a^ ^1^H NMR data were measured in CD_3_OD at 600 MHz

**Table 4:** ^13^C NMR spectroscopic data of compounds **5**–**7**

No.	5*^a^	6*^a^	7*^a^
1	162.2	162.2	161.9
2	117.4	117.5	117.5
3	146.7	146.8	147.7
4	121.9	121.9	121.2
5	113.0	110.9	113.3
6	166.1	165.4	164.1
7	108.1	107.8	106.3
8	162.0	161.8	161.7
9	188.4	188.6	188.6
10	57.3	57.6	57.3
1a	117.7	118.2	118.5
4a	139.8	140.6	141.9
5a	144.5	145.5	144.1
8a	116.5	117.8	116.1
3-Me	21.8	22.0	22.2
1′	162.9	162.9	162.0
2′	117.5	117.7	117.6
3′	147.5	147.6	147.7
4′	121.4	121.6	121.2
5′	111.7	112.6	111.0
6′	164.9	165.2	164.9
7′	104.2	106.2	105.5
8′	162.0	162.0	161.4
9′	188.4	188.3	188.5
10′	57.5	57.5	57.4
1a′	117.9	117.2	118.6
4a′	142.4	141.7	141.9
5a′	146.7	146.5	144.1
8a′	117.0	116.1	117.4
3′-Me	22.0	21.9	22.2
6′-OMe	56.5	56.4	56.1
1″	106.2	106.3	105.1
2″	74.9	75.0	75.1
3″	78.8	78.7	78.5
4″	71.6	71.4	71.6
5″	77.5	77.5	76.8
6″	62.9	62.8	62.7
1′′′	104.9	104.5	105.0
2′′′	74.5	74.5	75.1
3′′′	78.8	78.6	78.4
4′′′	71.2	71.3	71.3
5′′′	77.4	77.5	76.8
6′′′	62.6	62.6	62.6

^a^ ^13^C NMR data were measured in CD_3_OD at 150 MHz

### Polygonumnolide B2 (**6**)

Yellow powder; [α]_D_^25^ −110° (c = 0.10, MeOH); UV (MeOH) λ_max_ (logε): 208(4.81), 279 (4.41), 346 (4.38) nm; ECD (c = 1.15 × 10^−4^ M, MeOH): Δ*ε*_367.5 nm_ +3.45, Δ*ε*_300.0 nm_ −5.96; IR (KBr) ν_max_: 3382, 2918, 1630, 1599, 1492, 1353, 1334, 1295, 1217, 1177, 1076, 905, 864, 790 cm^−1^; ^1^H NMR (CD_3_OD, 600 MHz) and ^13^C NMR (CD_3_OD, 150 MHz) data, see Tables 3 and 4; HRESI-MS: *m/z* 847.2455 [M–H]^−^ (calcd for C_43_H_44_O_18,_ 847.2454).

### Polygonumnolide B3 (**7**)

Yellow powder; [α]_D_^25^ −230° (c = 0.10, MeOH); UV (MeOH) λ_max_ (logε): 208 (4.81), 279 (4.41), 346 (4.38) nm; ECD (c = 1.30 × 10^−4^ M, MeOH): Δ*ε*_376.0 nm_ +23.08, Δ*ε*_316.5 nm_ −47.53, Δ*ε*_279.0 nm_ +19.51; IR (KBr) ν_max_: 3382, 2918, 1630, 1599, 1492, 1353, 1334, 1295, 1217, 1177, 1076, 905, 864, 790 cm^−1^; ^1^H NMR (CD_3_OD, 600 MHz) and ^13^C NMR (CD_3_OD, 150 MHz) data, see Tables 3 and 4; HRESI-MS: *m/z* 847.2452 [M–H]^−^ (calcd for C_43_H_44_O_18,_ 847.2454).

### Enzyme hydrolysis of compounds

Compounds **1**–**7** (3.0 mg each) (Tian et al. 2013) were enzymatically hydrolyzed by β-glucosidase (10.0 mg) from almonds (CAS 9001-22-3) at 30 °C for 12 h, respectively. The reaction mixtures were extracted with EtOAC (3 × 10 mL). The aqueous layers were frozen for 12 h in order to remove organic solvent and freeze-dried to obtain the monosaccharides of **1**–**7**. l-Cysteine methyl ester hydrochloride (1.5 mg) was added to solutions of the monosaccharides of compounds **1**–**7** and d-glucose in pyridine (3.0 mL) and kept at 60 °C for 1 h. The reaction mixtures were cooled in an ice-water bath, trimethylsilylimidazole (1.0 mL) was added and the mixtures heated to 60 °C for 30.0 min. The reaction mixtures were partitioned between H_2_O (2 mL) and *n*-hexane (3 × 10 mL). The *n*-hexane extracts of each digest were subjected to GC analysis, run on a Agilent 7890A gas chromatograph equipped with a Agilent HP-5 capillary column (60.0 m × 0.32 mm × 1.0 µm) and an H_2_ flame ionization detector with the following conditions: column temperature, 160–280 °C; ramp, 5 °C/min and maintained at 280 °C for 20.0 min; carrier gas, N_2_ (1 mL/min); injector and detector temperature, 300 °C; injection volume, 5.0 µL; and split ratio, 1/30. The configuration of the monosaccharides in each sample was determined by comparing the retention time of the derivatives with that of an authentic sample. All the samples gave a single peak with the same retention time of 40.6 min and carbohydrates in **1**–**7** were determined to all be d-glucose.

### Cytotoxicity assay

A tetrazolium-based colorimetric assay (methyl thiazolyl tetrazolium assay, MTT assay) was used to assess the cytotoxicity of **1**–**7** against KB tumor cell lines with taxol used as the positive control. The assays were performed according to a published technique (Zhang et al. 2001).

## Results and discussion

**Fig. 1:**
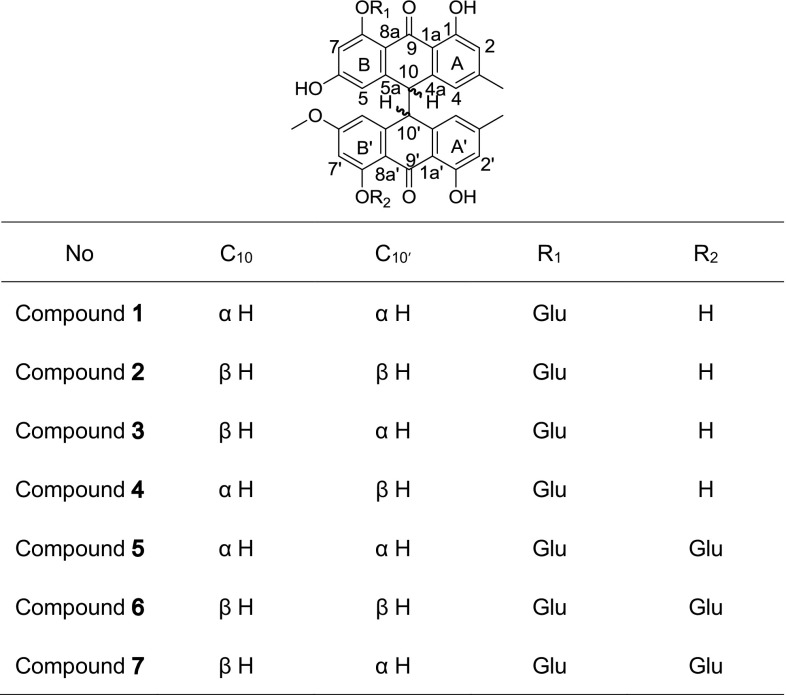
Structures of compounds **1**–**7** from the roots of *Polygonum multiflorum* Thunb

**Fig. 2:**
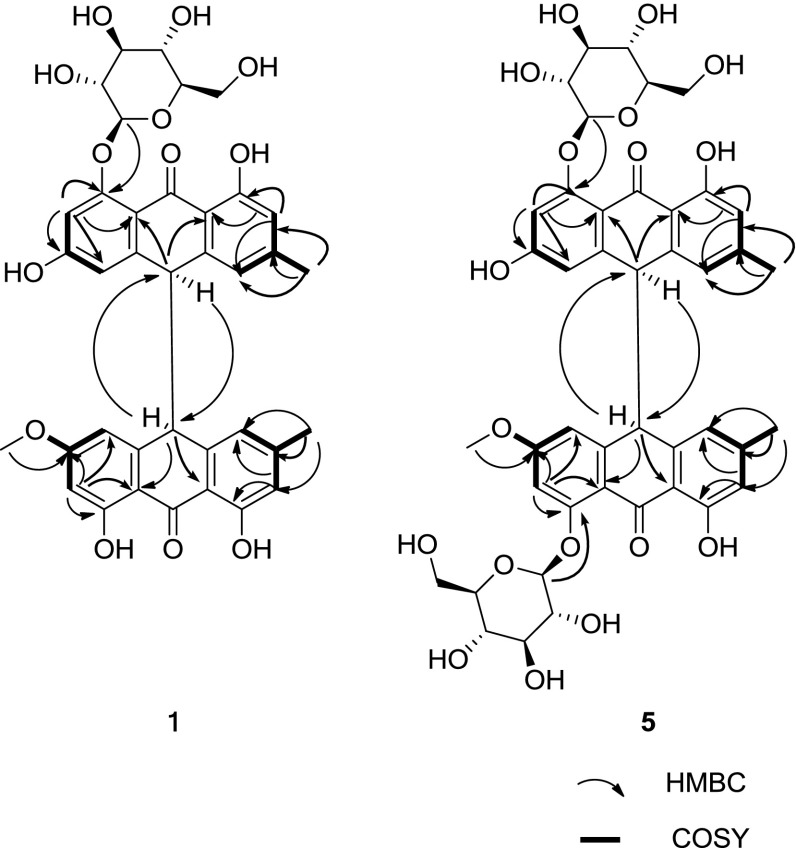
Key ^1^H–^1^H COSY and HMBC correlations of compounds **1** and **5**

Compound **1** (Fig. 1) was obtained as a yellow powder. Its molecular formula, C_37_H_34_O_13_, was deduced from HRESI-MS from the peak at *m/z* 685.1940 [M–H]^−^, which corresponded to 21 indices of hydrogen deficiency. The IR spectrum showed strong absorption bands at 1620 and 1599 cm^−1^ that were assigned to carbonyl groups, a peak from chelated hydroxyl groups at 3361 cm^−1^ and a peak from aromatic ring functionalities at 1489 cm^−1^. The UV spectrum showed absorption maxima at 207, 280 and 351 nm, which were very close to those of previously reported dianthrone derivatives (Lemli et al. 1964; Vandenberg and Labadie 1981; Gizachew et al. 1993). The ^1^H NMR (Table 1) and ^1^H–^1^H COSY spectra (Fig. 2) displayed the signals of eight meta-coupled aromatic protons [*δ*_H_ 6.45 (1H, s, H-2), 5.58 (1H, br s, H-4), 6.30 (1H, br s, H-5), 6.83 (1H, d, *J* = 2.0 Hz, H-7), 6.59 (1H, s, H-2′), 6.34 (1H, br s, H-4′), 5.45 (1H, br s, H-5′), and 6.18 (1H, d, *J* = 2.0 Hz, H-7′)], two methyl groups [*δ*_H_ 2.07 (3H, s, Me-3) and 2.29 (3H, s, Me-3′)], one methoxy group at *δ*_H_ 3.74 (3H, s, OCH_3_-6′), two vicinal methine protons [*δ*_H_ 4.14 (1H, d, *J* = 3.5 Hz, H-10) and 4.08 (1H, d, *J* = 3.5 Hz, H-10′)], and one β-glucopyranosyl anomeric proton at *δ*_H_ 4.60 (1H, d, *J* = 7.5 Hz, H-1′′).

Comparison of the 1D NMR spectrum of **1** with those of physcion dianthrones, emodin dianthrones, and physcion-emodin dianthrones (Donald et al. 1976; Du et al. 2008; Monache et al. 1991) suggested **1** could be a C-10/C-10′ isomer of physcion-emodin dianthrone glycosides. The ^13^C NMR and DEPT spectra (Table 2) exhibited 37 carbon signals, including 31 carbon signals of physcion-emodin dianthrones that were categorized by DEPT and HSQC techniques into 18 quaternary carbons, including with two carbonyl groups [*δ*_C_ 191.3 (C-9′) and 188.5 (C-9)], 10 methine groups [*δ*_C_ 117.3 (C-2), 121.8 (C-4), 112.5 (C-5), 107.7 (C-7), 117.7 (C-2′), 122.2 (C-4′), 109.7 (C-5′), 100.7 (C-7′), 57.7 (C-10) and 56.7 (C-10′)], two methyl groups [*δ*_C_ 21.8 (Me-3) and 22.1 (Me-3′)], one methoxy group *δ*_C_ 56.5 (OMe-6′)] and six carbon signals characteristic of glucose [*δ*_C_ 106.1, 74.9, 78.8, 71.2, 77.4, 62.7].

The HMBC spectrum (Fig. 2) of **1** showed long-range correlations from H-2 (*δ*_H_ 6.45) to C-1 (*δ*_C_ 162.3), C-4 (*δ*_C_ 121.8), and C-1a (*δ*_C_ 117.5), and from Me-3 (*δ*_H_ 2.07) to C-2 (*δ*_C_ 117.3), C-3 (*δ*_C_ 146.8), and C-4 (*δ*_C_ 121.8), indicating the presence of an A ring. The HMBC correlations between H-7 (*δ*_H_ 6.83) and C-5 (*δ*_C_ 112.5), C-6 (*δ*_C_ 164.6), C-8 (*δ*_C_ 161.9), and C-8a (*δ*_C_ 117.1) indicated the presence of a B ring. Meanwhile, the HMBC correlations from H-2′ (*δ*_H_ 6.59) to C-1′ (*δ*_C_ 162.6), C-4′ (*δ*_C_ 122.2), and C-1a′ (*δ*_C_ 116.0), and from Me-3′ (*δ*_H_ 2.29) to C-2′ (*δ*_C_ 117.7), C-3′ (*δ*_C_ 148.3), and C-4′ (*δ*_C_122.2) indicated the presence of an A′ ring. The HMBC correlations from H-7′ (*δ*_H_ 6.18) to C-5′ (*δ*_C_ 109.7), C-6′ (*δ*_C_ 166.4), C-8′ (*δ*_C_ 165.7), and C-8a′ (*δ*_C_ 111.5), and from OCH_3_-6′ to C-6′ (*δ*_C_ 166.4) indicated the presence of an B′ ring. The configuration of the C-10/C-10′ junction of the two anthronyl moieties was deduced from the HMBC correlations observed between the proton at *δ*_H_ 4.14 (1H, d, *J* = 3.5 Hz, H-10) and C-1a (*δ*_C_ 117.5), C-4 (*δ*_C_ 121.8), C-5 (*δ*_C_ 112.5), C-8a (*δ*_C_ 117.1), and C-10′ (*δ*_C_ 56.5) and between the proton at *δ*_H_ 4.08 (1H, d, *J* = 3.5 Hz, H-10′) and C-1a′ (*δ*_C_ 116.0), C-4′ (*δ*_C_ 122.2), C-5′ (*δ*_C_ 109.7), C-8a′ (*δ*_C_ 111.5), and C-10 (*δ*_C_ 57.7). The HMBC correlations between *δ*_H_ 4.60 (1H, d, *J* = 7.5 Hz, H-1′′) and C-8 (*δ*_C_ 161.9) suggested that the sugar moiety in **1** was attached at C-8. The β-pyranoside configuration was inferred from the coupling constant (^3^ *J*_H-1′′, H-2′′_ = 7.5 Hz) (Tian et al. 2013), while the d-glucosyl stereochemistry was determined on the basis of the enzymatic hydrolysis with β-glycoside hydrolase, followed by GC analysis of its corresponding trimethylsilylated l-cysteine adduct. Thus, the planar structure of **1** was assigned as physcion-emodin-8-*O*-β-d-glucopyranoside dianthrone.

The relative structure of dianthrone derivatives was unable to be confirmed by NOESY experiments (Lenta et al. 2008; Haasnoot et al. 1980; Spassov 1971). Indeed, the ROESY spectrum correlations of H-10 with H-4, H-5, H-4′ and H-5′, and H-10′ with H-4, H-5, H-4′ and H-5′ indicated that the relative structure of **1** cannot be confirmed by NOESY. Three of the four C-10/10′ diastereomers of prinoidin–emodin dianthrones (Mai et al. 2001) isomers gave two doublets (*J* = 3.0 Hz) for the *δ*_H_ H-10 and H-10′ signals. Thus, the relative structure of dianthrone derivatives cannot be confirmed from the coupling constants between H-10 and H-10′.

The anti-conformer configuration is suggested to be neglected relative to two predominant gauche conformers with crossed rings A/B′ (I) and A′/B (II) for the *cis* H-10/10′ dianthrones or crossed A/A′ (III) and B/B′ (IV) for the *trans* H-10/10′ dianthrones (Angela et al. 2007; Ji et al. 2014). The crossed rings A/B′ (I), A′/B (II), A/A′ (III) and B/B′ (IV) shift ^1^H NMR peaks form the overlapped parts upfield because of their mutually-shielding effect. The H-4′/H-5 and H-4/H-5′ peaks in the crossed rings A/B′ (I) and A′/B (II) of *cis* H-10/10′ dianthrones shift upfield because of the shielding effects from A′/B and A/B′, respectively. The H-4′/H-4 and H-5′/H-5 peaks from the crossed rings A/A′ (III) and B/B′ (IV) of *trans* H-10/10′ dianthrones shift upfield because the shielding effect from A/A′ and B/B′, respectively. On the basis of above evidences, the H-4 (*δ*_H_ 5.58) and H-5′ (*δ*_H_ 5.45) peaks of **1** are more upfield than the H-5 (*δ*_H_ 6.30) and H-4′ (*δ*_H_ 6.34) peaks due to the shielding effect from A′/B (II) rings, suggesting the relative structure of **1** was confirmed as a *cis* H-10/10′ dianthrone (Fig. 1).

Compounds **2**–**4** (Fig. 1) had the same molecular formula C_37_H_34_O_13_ as **1**, as determined by HRESI-MS, corresponding to 21 degrees of unsaturation. The IR, UV and NMR spectra of **2**–**4** (Tables 1, 2) were similar with those of **1**. The structures were deduced from 1D NMR and 2D NMR spectra, as well as the comparison of their NMR data with those of **1**. The signals of H-10 and H-10′ differed from **2** to **4**. In the ^1^H NMR spectra of **1**, **2** and **4**, the H-10 and H-10′ signals all appeared as two doublets at *δ*_H_ 4.14 (1H, d, *J* = 3.5 Hz, H-10) and 4.08 (1H, d, *J* = 3.5 Hz, H-10′) (**1**); *δ*_H_ 4.57 (1H, d, *J* = 3.6 Hz, H-10) and 4.56 (1H, d, *J* = 3.6 Hz, H-10′) (**2**); and *δ*_H_ 4.39 (1H, d, *J* = 3.0 Hz, H-10) and 4.35 (1H, d, *J* = 3.0 Hz, H-10′) (**4**). The ^1^H NMR spectrum of **3** revealed a 2H singlet assigned to both these protons at *δ*_H_ 4.55 (2H s, H-10, 10′) (**3**). In the HMBC spectra of **1**–**4**, the correlations from H-1″ to C-8 and from OCH_3_-6′ to C-6′ were observed, suggesting that **1**–**4** were four C-10/C-10′ diastereomers of physcion-emodin-8-*O*-glucopyranoside dianthrone. A β-configuration was inferred for these compounds from the anomeric coupling constants (^3^ *J*_H-1″, H-2″_ >7.0 Hz), while the d-glucosyl absolute configuration was determined from enzymatic hydrolysis with β-glucosidase hydrolase. The planar structures of **2**–**4** were elucidated as emodin–emodin-8-*O*-β-d-glucopyranoside dianthrones.

The H-4/H-5′ signals of **1** and **2** are shifted to downfield due to the shielding effect from A′/B (II) rings, respectively. The H-4′/H-4 signals of **3** and **4** are also shifted to downfield due to the shielding effect from A/A′ (III) rings, respectively. These data suggest the relative structures of **1** and **2** were *cis* H-10/10′ dianthrones, while the relative structures of **3** and **4** were *trans* H-10/10′ dianthrones. Compounds **1**–**4** were named as polygonumnolide A1, A2, A3 and A4, respectively.

Compounds **5**–**7** (Fig. 1) were obtained as yellow powders. They had the same molecular formula C_43_H_44_O_18_, as determined by HRESI-MS, corresponding to 22 degrees of unsaturation. The IR, UV and NMR spectra of **5**–**7** (Tables 3, 4) were similar with those of **1**–**4**, except they showed additional peaks assigned to an additional glucose, which indicated that **5**–**7** may be three C-10/C-10′ diastereomers of physcion-8-*O*-β-d-glucopyranoside-emodin-8-*O*-β-d-glucopyranoside dianthrones. The structures of these compounds were deduced from 1D NMR spectra, such as ^1^H NMR (Fig. 2), and 2D NMR spectra, such as ^1^H-^1^H COSY, HSQC and HMBC (Fig. 2), as well as the comparison of their NMR data with that of **1**–**4.** The ^1^H NMR signals of H-10 and H-10′ differed from **5** to **7**, appearing as two different signals at *δ*_H_ 4.30 (1H, s, H-10) and 4.32 (1H, s, H-10′) (**5**), *δ*_H_ 4.39 (1H, d, *J* = 3.0 Hz, H-10) and 4.32 (1H, *J* = 3.0 Hz, H-10′) (**6**), and a 2H singlet at *δ*_H_ 4.25 (2H, s, H-10, H-10′) (**7**). The HMBC correlations from H-1′′ to C-8, H-1′′′ to C-8′ and from OCH_3_-6′ to C-6′ in the spectra of **5**–**7** suggested these compounds were three C-10/C-10′ diastereomers of physcion-8-*O*-glucopyranoside-emodin-8-*O*-glucopyranoside dianthrones. Furthermore, the anomeric centers were all assigned as β-configurations from the coupling constants (^3^ *J*_H-1″, H-2″_ = 7.2 Hz and ^3^ *J*_H-1′′′, H-2′′′_ = 7.8 Hz) (**5**), (^3^ *J*_H-1″, H-2″_ = 7.8 Hz and ^3^ *J*_H-1′′′, H-2′′′_ = 7.2 Hz) (**6**) and (^3^ *J*_H-1″, H-2″_ = 7.8 Hz and ^3^ *J*_H-1′′′, H-2′′′_ = 7.2 Hz) (**7**). Enzymatic hydrolysis of these compounds with β-glucosidase hydrolase confirmed all the sugars were d-glucose. Thus, the planar structures of **5**–**7** were inferred as physcion-8-*O*-β-d-glucopyranoside-emodin-8-*O*-β-d-glucopyranoside dianthrones.

The H-4/H-5′signals of **5** and **6** are shifted to downfield due to the shielding effect from A′/B (II) rings, respectively. Additionally, the H-5′/H-5 signals of **7** are shifted to downfield due to the shielding effect from B/B′ (IV) rings. These data suggest the relative structures of **5** and **6** were *cis* H-10/10′ dianthrones, while the relative structure of **7** was a *trans* H-10/10′ dianthrone. Compounds **5**–**7** were named as polygonumnolide B1, B2 and B3, respectively.

Compounds **1**–**7** were bioassayed for cytotoxicity activity against KB tumor cell lines using taxol as the positive control (Zhang et al. 2001). Compounds **1**–**4** exhibited moderate cytotoxicities against the KB cell lines (Table 5).

**Table 5:** Cytotoxic activities of compounds (**1**–**7**) against KB human epidermoid cancer cell lines by the MTT method

Compound	IC_50_^a^ (μm)
Taxol^b^	0.53
**1**	29.7
**2**	35.6
**3**	36.8
**4**	31.1
**5**	82.4
**6**	88.6
**7**	95.8

^a^IC_50_ value of compounds against KB human epidermoid cancer cell lines, which was defined as the concentration (μm) that caused 50 % inhibition of cell growth in vitro

^b^Taxol as a positive control

## Electronic supplementary material

Below is the link to the electronic supplementary material.

Spectroscopic data for the new compounds (**1–7**) including ^1^H, ^13^C, and 2D NMR, IR and ESI-MS/MS data are provided in the supporting information
