# Correction to: Polygonumnolides A1–B3, minor dianthrone derivatives from the roots of *Polygonum multiflorum* Thunb

**DOI:** 10.1007/s12272-018-1092-5

**Published:** 2018-12-19

**Authors:** Jianbo Yang, Zheng Yan, Jin Ren, Zhong Dai, Shuangcheng Ma, Aiguo Wang, Yalun Su

**Affiliations:** 1 State Key Laboratory of Bioactive Substance and Function of Natural Medicines, Institute of Materia Medica, Chinese Academy of Medical Sciences and Peking Union Medical College, Beijing, 100050 People’s Republic of China; 2 Research and Inspection Center of Traditional Chinese Medicine and Ethnomedicine, National Institutes for Food and Drug Control, State Food and Drug Administration, Beijing, 100050 People’s Republic of China; 3 School of Chinese Pharmacy, Beijing University of Chinese Medicine, Beijing, 100102 People’s Republic of China

## Correction to: Arch. Pharm. Res. (2018) 41:617–624 10.1007/s12272-016-0816-7

The author would like to include conflict of interest statement and Grant Numbers (81773874 and 81703665) in the acknowledgement section of the online published article. The conflict of interest statement and acknowledgement section should read as:

### **Conflict of interest**

The authors declare that there are no conflicts of interest.

### **Acknowledgements**

The authors are grateful to the members of the analytical group of the State Key Laboratory of Bioactive Substance and Function of Natural Medicines, Institute of Materia Medica, Chinese Academy of Medical Sciences and Peking Union Medical College, Beijing, China, for measuring the spectroscopic data. This Project was financially supported by the National Natural Science Foundation of China (Grant Nos. 81173506, 81773874 and 81703665) and the 12th 5 Year National significant new drugs creation feature subjects-traditional Chinese medicine quality safety evaluation and risk control technology platform (2014ZX09304307-002).

