# Supplementary material for: Polygonumnolides A1–B3, minor dianthrone derivatives from the roots of Polygonum multiflorum Thunb
Source: Arch Pharm Res. 2017 Jul 5;41(6):617–24. doi: 10.1007/s12272-016-0816-7 (PMC6028828; doi:10.1007/s12272-016-0816-7)

**Polygonumnolides A1**–**B3, minor dianthrone derivatives from the roots of *Polygonum multiflorum* Thunb**

**Jianbo Yang**^1, 2, 3^**. Zheng Yan**^1^**. Jin Ren**^1^**. Zhong Dai**^2^**. Shuangcheng Ma**^2, 3^***. Aiguo Wang**^1^***. Yalun Su**^1^

1 State Key Laboratory of Bioactive Substance and Function of Natural Medicines, Institute of Materia Medica, Chinese Academy of Medical Sciences and Peking Union Medical College, Beijing 100050, People′s Republic of China.

2 Research and Inspection Center of Traditional Chinese Medicine and Ethnomedicine, National Institutes for Food and Drug Control, State Food and Drug Administration,  Beijing 100050, People′s Republic of China.

3 School of Chinese Pharmacy, Beijing University of Chinese Medicine, Beijing 100102, People′s Republic of China.

Corresponding Author

*Corresponding author. Tel: 0086-10-67095272. Fax: 0086-10-67095887.

Email: [masc@nifdc.org.cn](mailto:masc@nifdc.org.cn) and wangaiguo@imm.ac.cn

**Content List**

| N0 | Content | pages |
| --- | --- | --- |
| Compound **1** | IR  HRESIMS  ^1^H NMR  ^13^C NMR  DEPT  ^1^H-^1^H COSY  HSQC  HMBC  ROESY | 5  6  7  8  9  10  11  12  13 |
| Compound **2** | IR  HRESIMS  ^1^H NMR  ^13^C NMR  DEPT  ^1^H-^1^H COSY  HSQC  HMBC | 14  15  16  17  18  19  20  21 |
| Compound **3** | IR  HRESIMS  ^1^H NMR  ^13^C NMR  DEPT  ^1^H-^1^H COSY  HSQC  HMBC | 22  23  24  25  26  27  28  29 |
| Compound **4** | IR  HRESIMS  ^1^H NMR  ^13^C NMR  DEPT  ^1^H-^1^H COSY  HSQC  HMBC  ROESY | 30  31  32  33  34  35  36  37  38 |
| Compound **5** | IR  HRESIMS  ^1^H NMR  ^13^C NMR  DEPT  ^1^H-^1^H COSY  HSQC  HMBC  ROESY | 39  40  41  42  43  44  45  46  47 |
| Compound **6** | IR  HRESIMS  ^1^H NMR  ^13^C NMR  DEPT  ^1^H-^1^H COSY  HSQC  HMBC  ROESY | 48  49  50  51  52  53  54  55  56 |
| Compound **7** | IR  HRESIMS  ^1^H NMR  ^13^C NMR  DEPT  ^1^H-^1^H COSY  HSQC  HMBC | 57  58  59  60  61  62  63  64 |

Polygonumnolide A1. IR spectrum of the new compound **1**


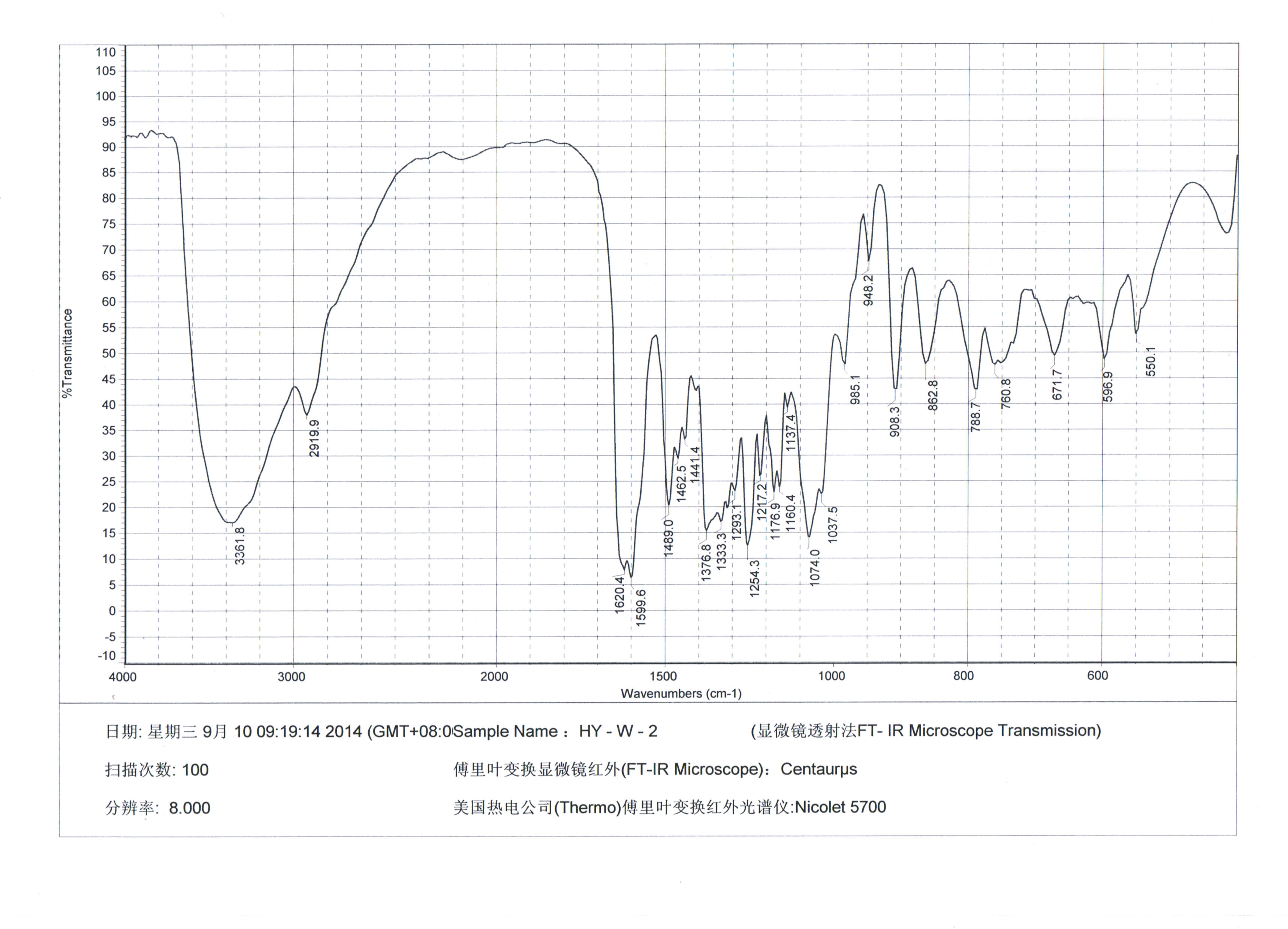


Polygonumnolide A1. HRESIMS spectrum of the new compound **1**


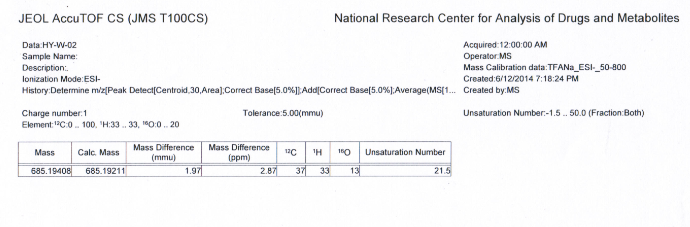


Polygonumnolide A1. ^1^H NMR (600 MHz, CD_3_OD) spectrum of the new compound **1**
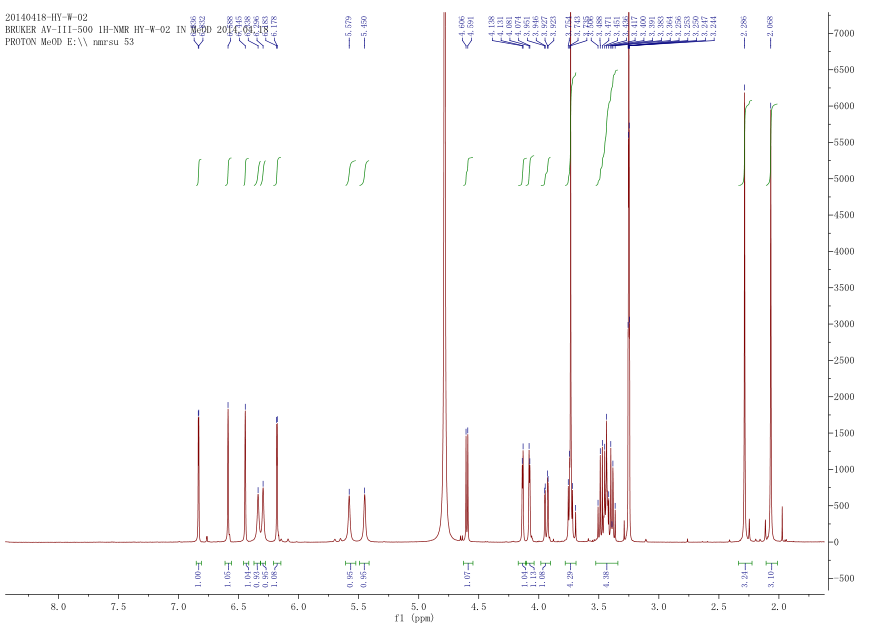


Polygonumnolide A1. ^13^C NMR (150 MHz, CD_3_OD) spectrum of the new compound **1
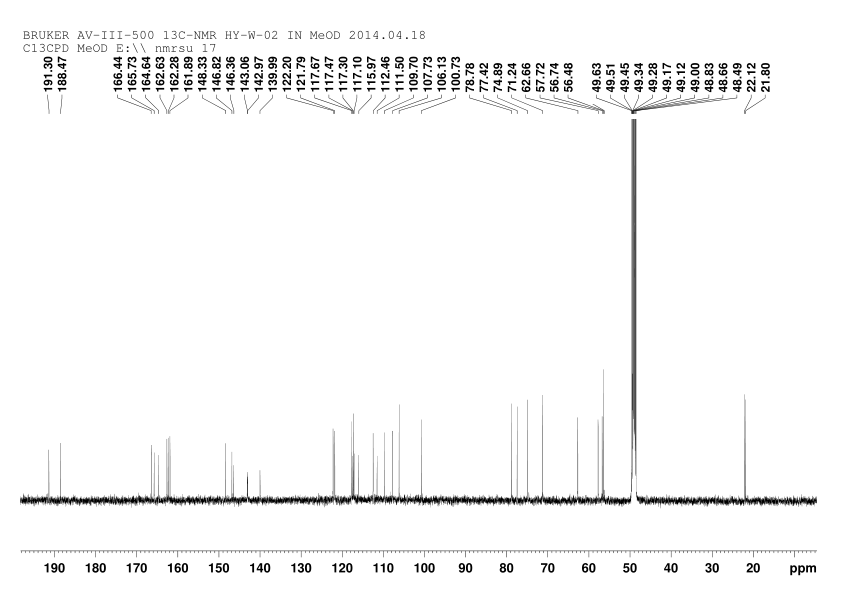
**

Polygonumnolide A1. DEPT spectrum of the new compound **1**


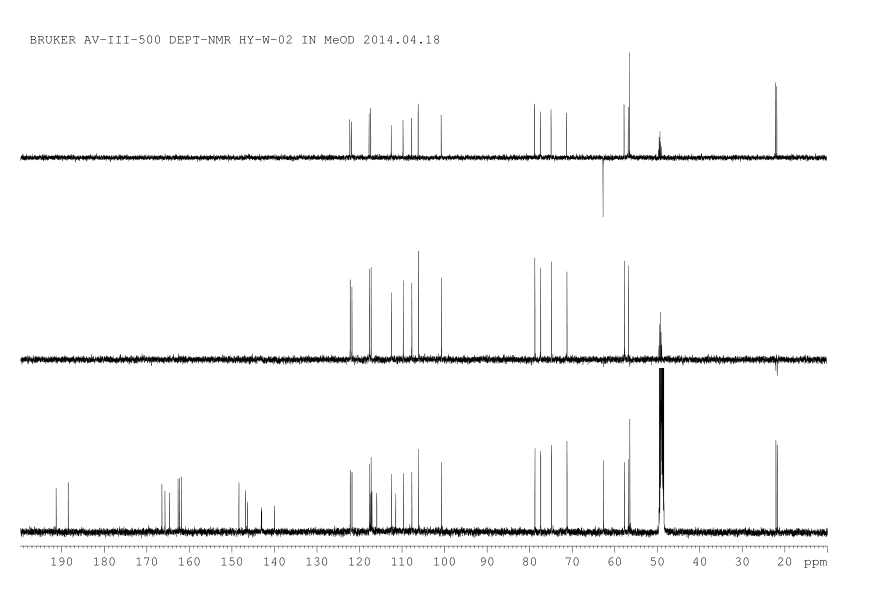


Polygonumnolide A1. ^1^H-^1^H COSY spectrum of the new compound **1**


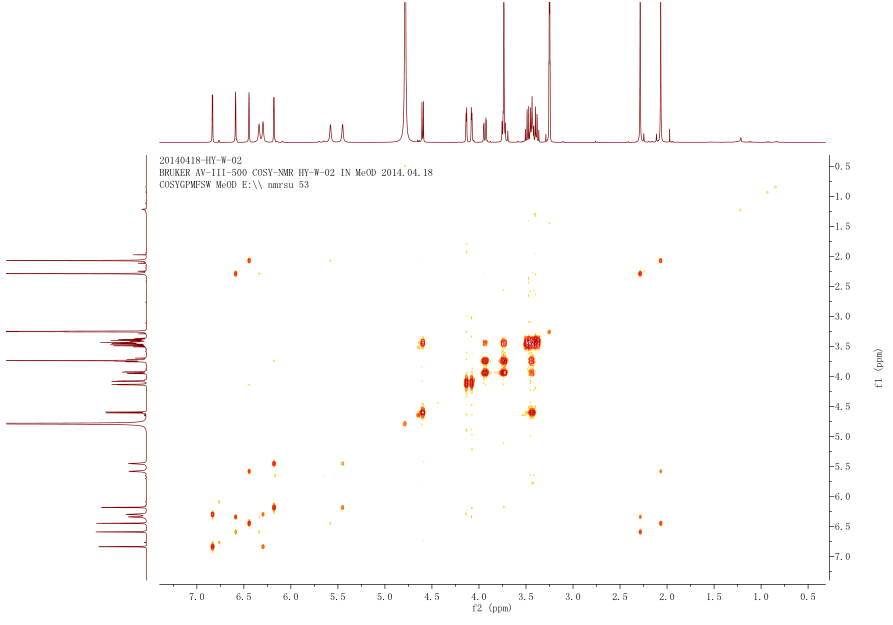


Polygonumnolide A1. HSQC spectrum of the new compound **1**


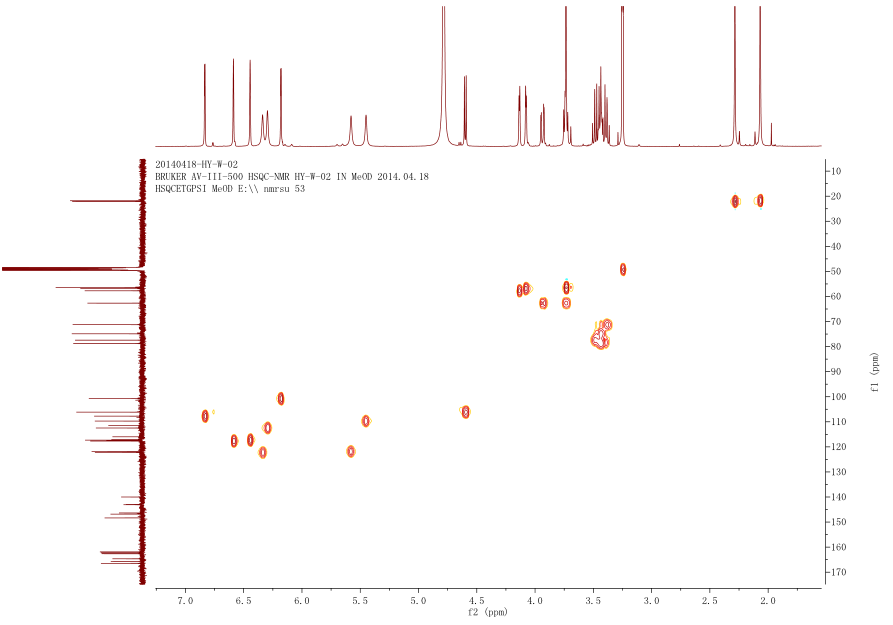


Polygonumnolide A1. HMBC spectrum of the new compound **1**


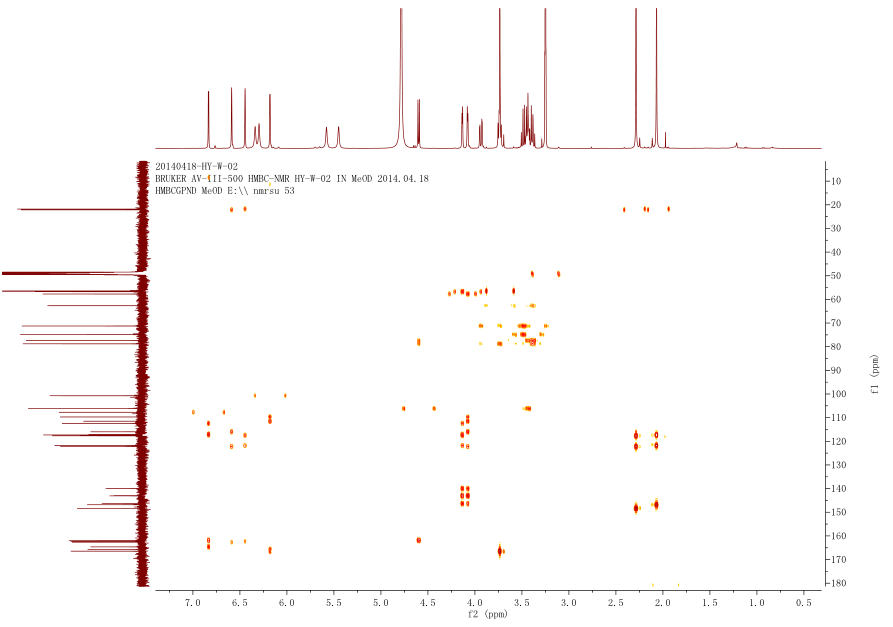


Polygonumnolide A1. ROESY spectrum of the new compound **1**


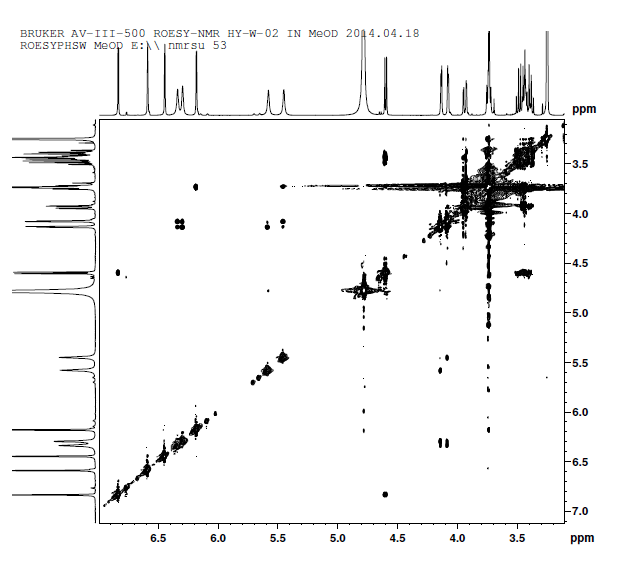


Polygonumnolide A2. IR spectrum of the new compound **2**


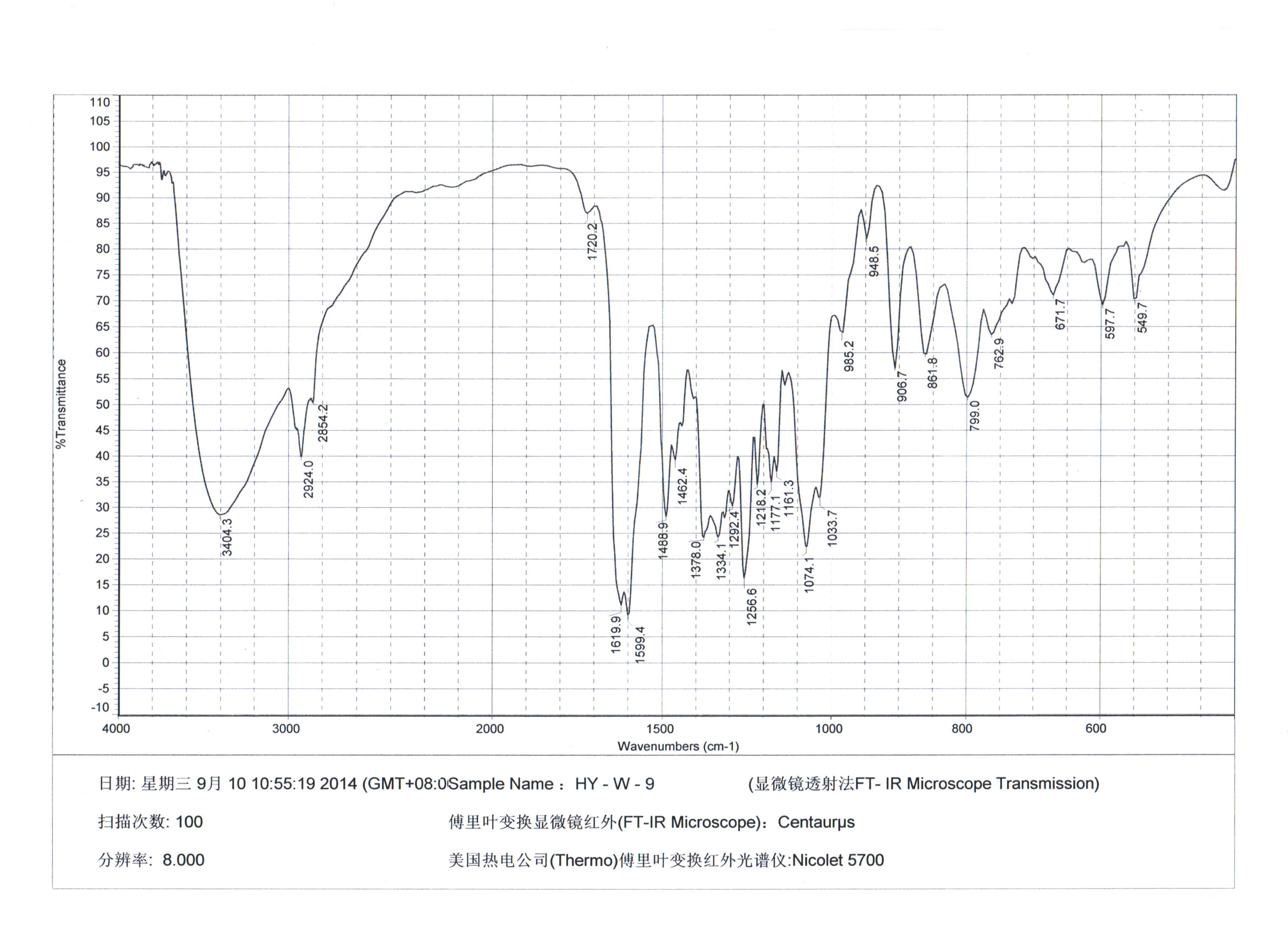


Polygonumnolide A2. HRESIMS spectrum of the new compound **2**


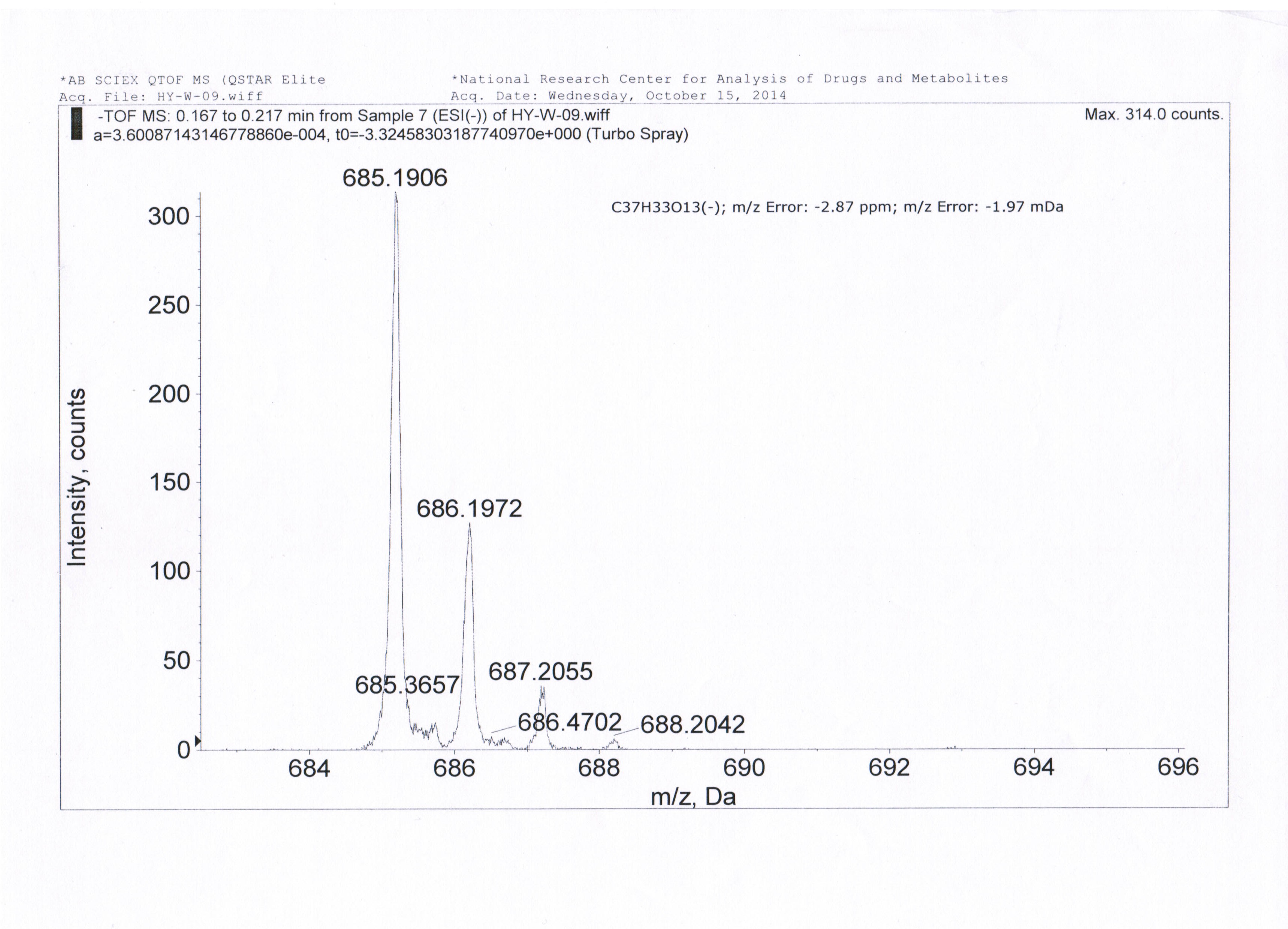


Polygonumnolide A2. ^1^H NMR (600 MHz, CD_3_COCD_3_) spectrum of the new compound **2**


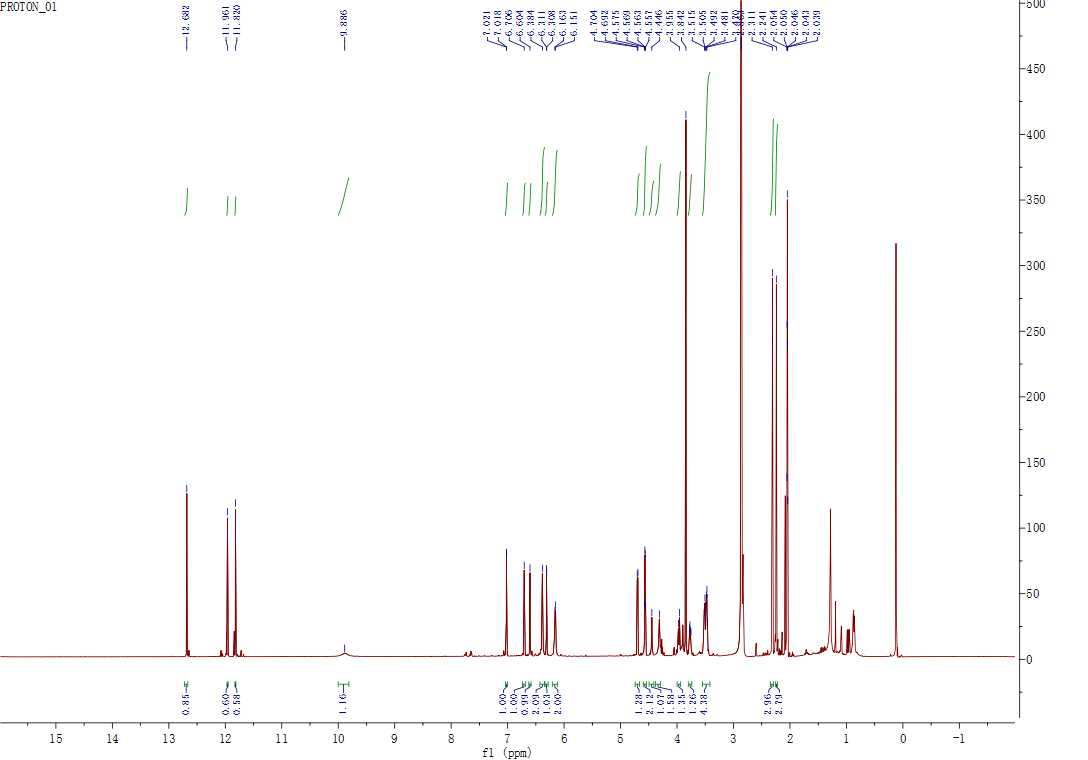


PolygonumnolideA2. ^13^C NMR (150 MHz, CD_3_COCD_3_) spectrum of the new compound **2**


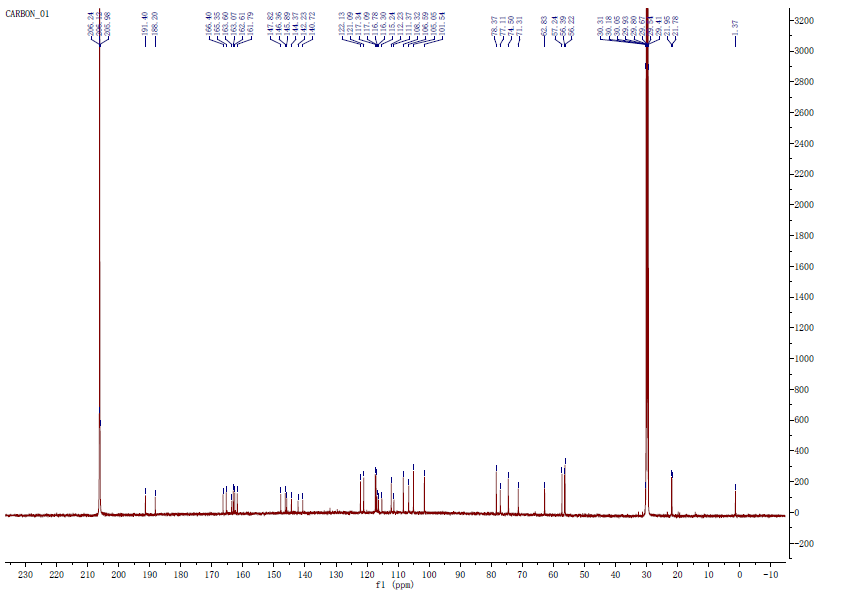


PolygonumnolideA2. DEPT spectrum of the new compound **2**


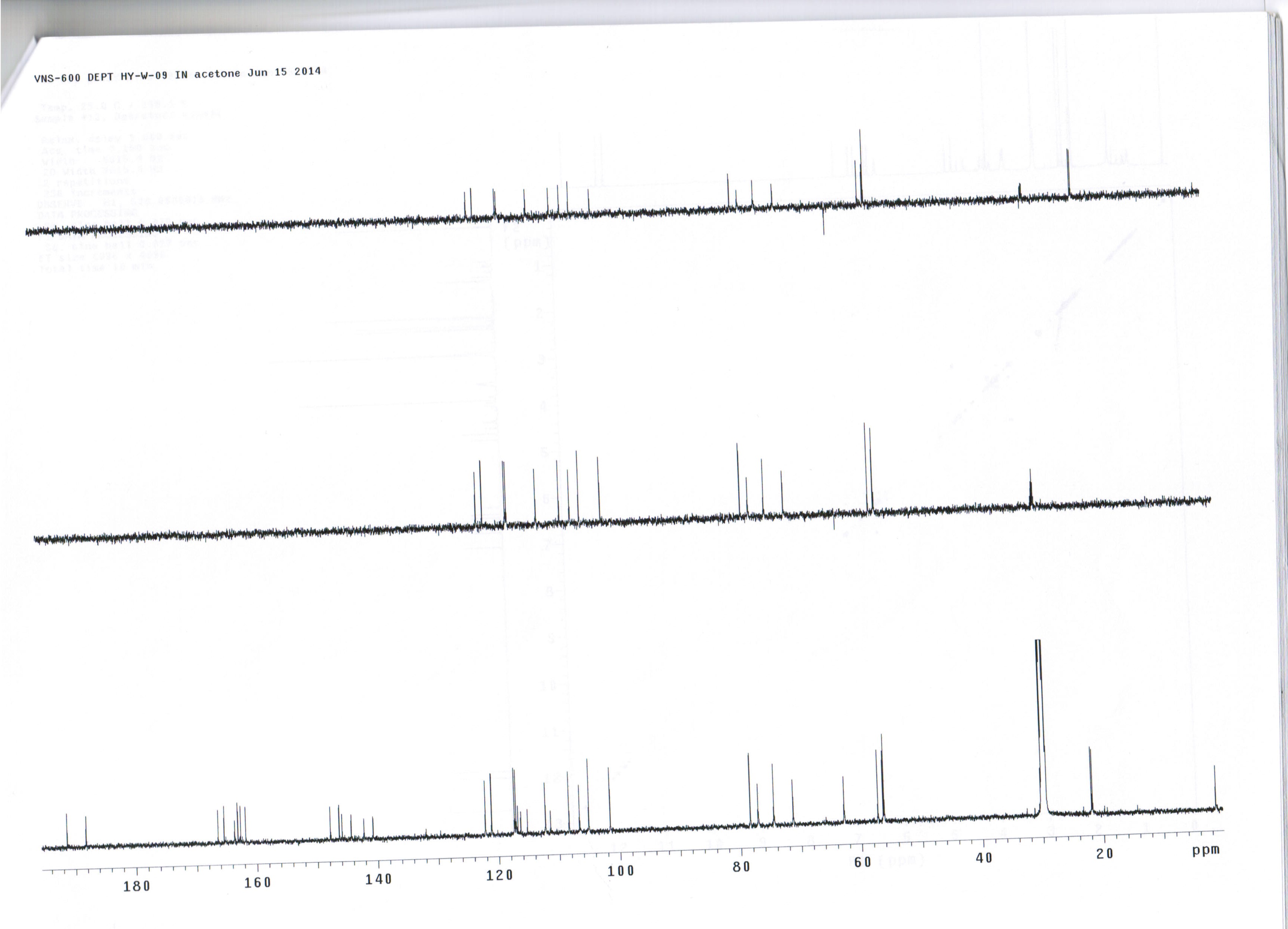


Polygonumnolide A2. ^1^H-^1^H COSY spectrum of the new compound **2**


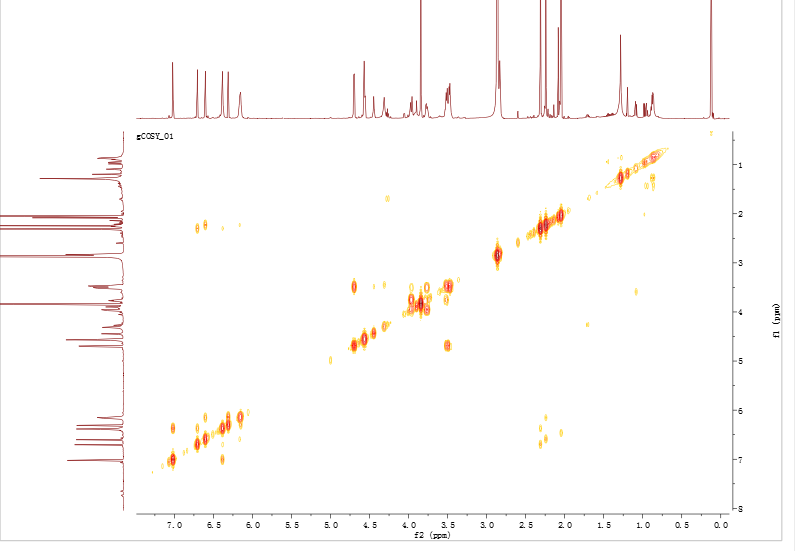


Polygonumnolide A2. HSQC spectrum of the new compound **2**


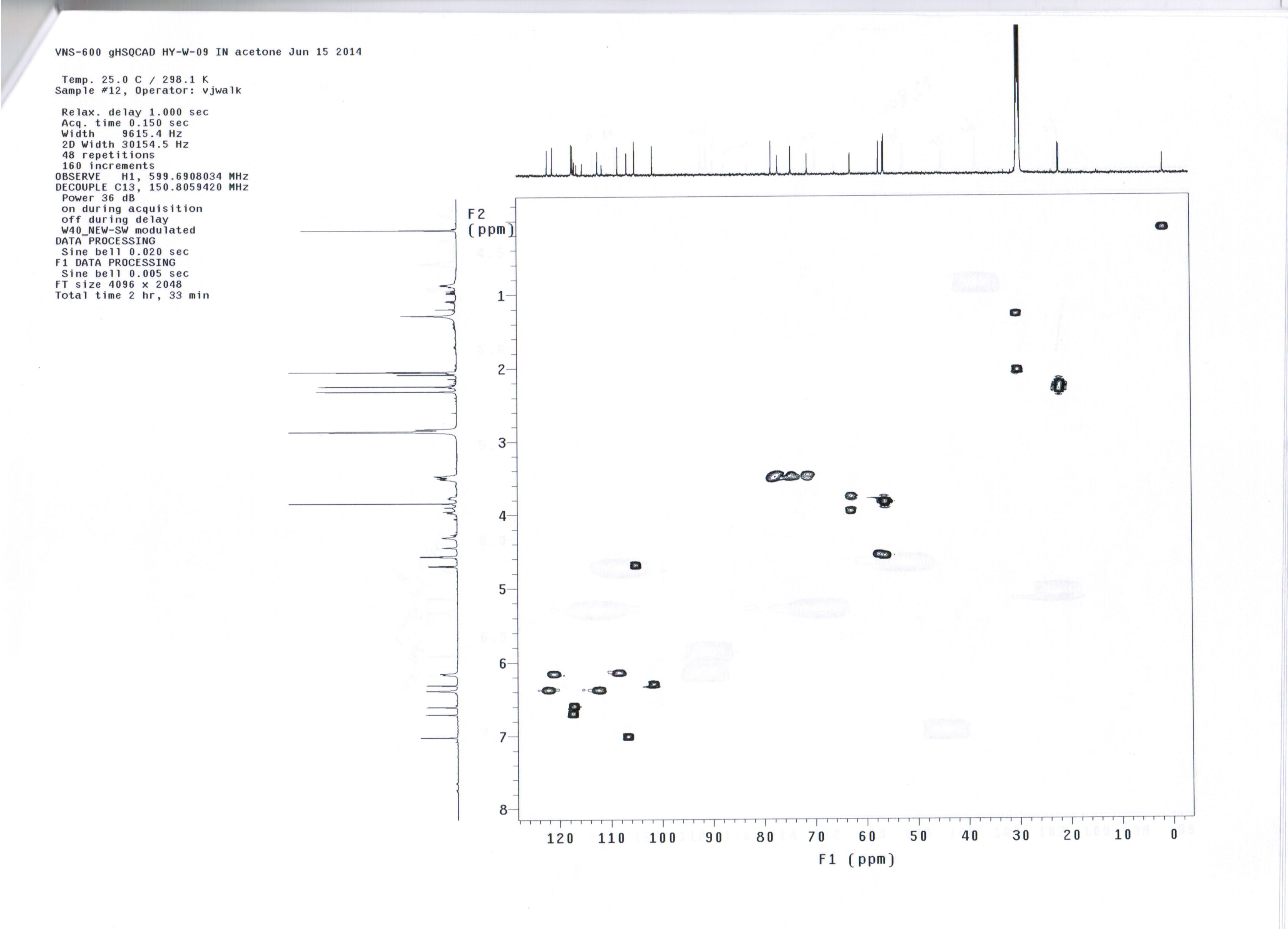


Polygonumnolide A2. HMBC spectrum of the new compound **2**


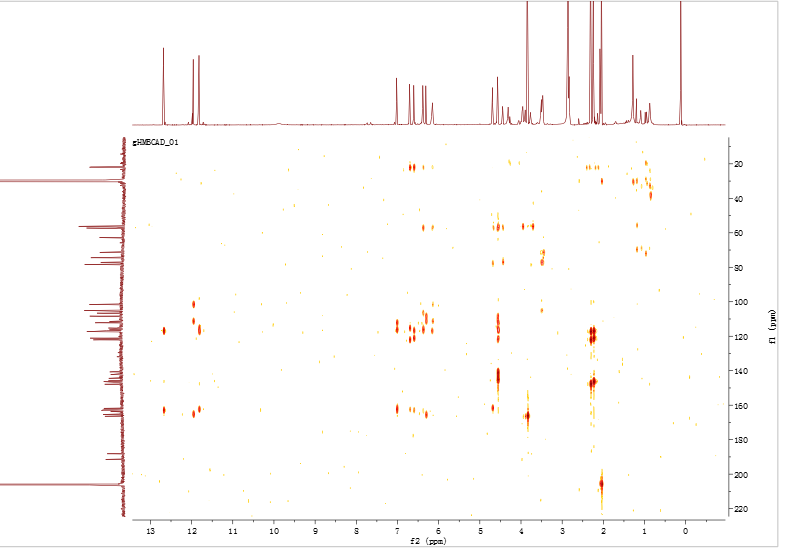


Polygonumnolide A3. IR spectrum of the new compound **3**


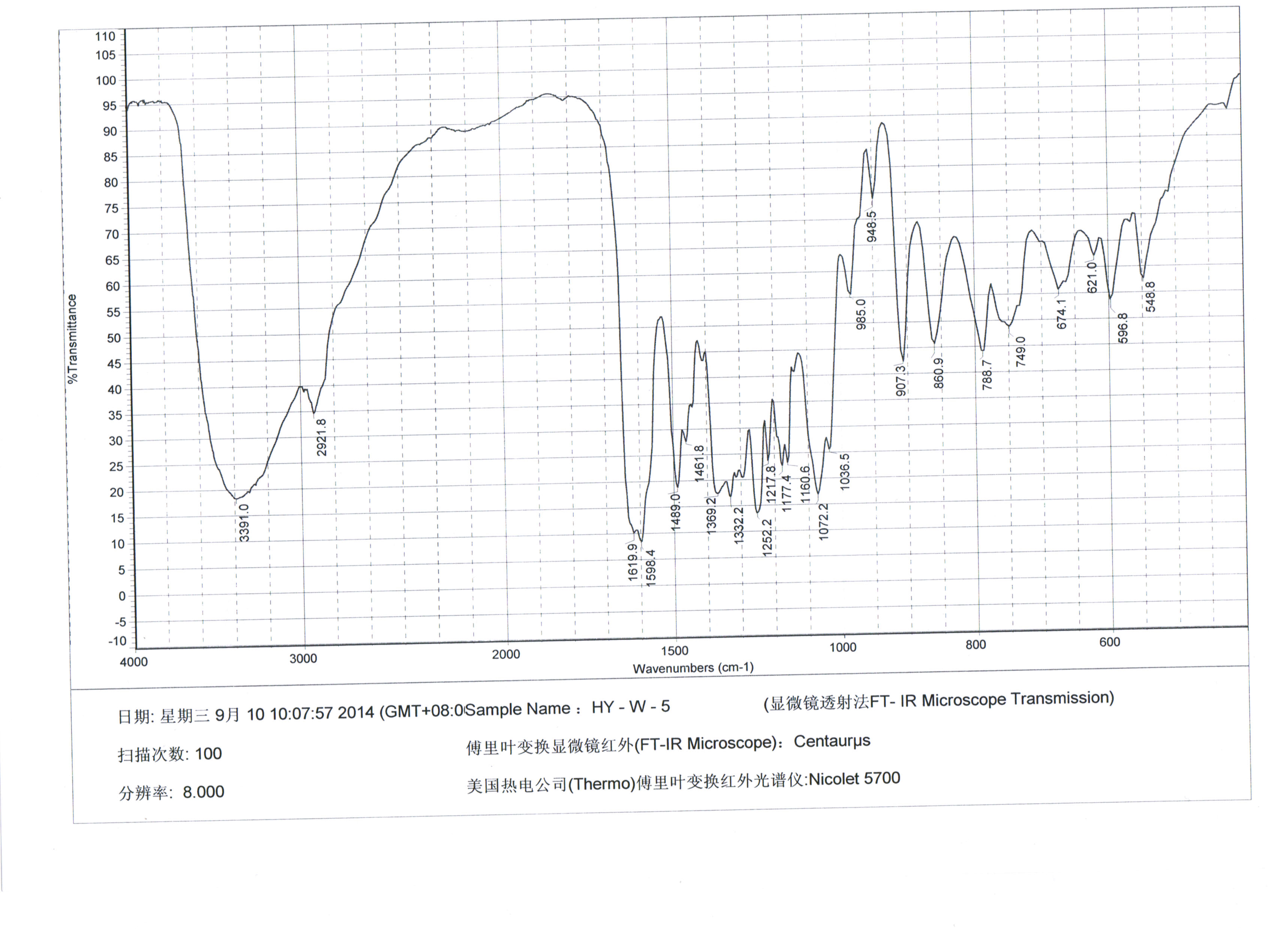


Polygonumnolide A3. HRESIMS spectrum of the new compound **3**


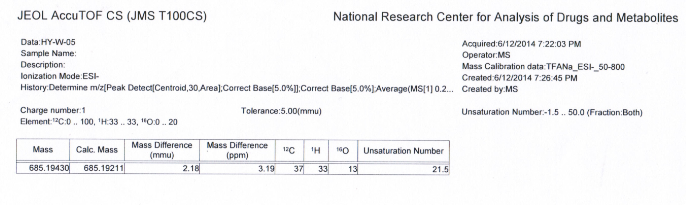


Polygonumnolide A3. ^1^H NMR (600 MHz, CD_3_COCD_3_) spectrum of the new compound **3**


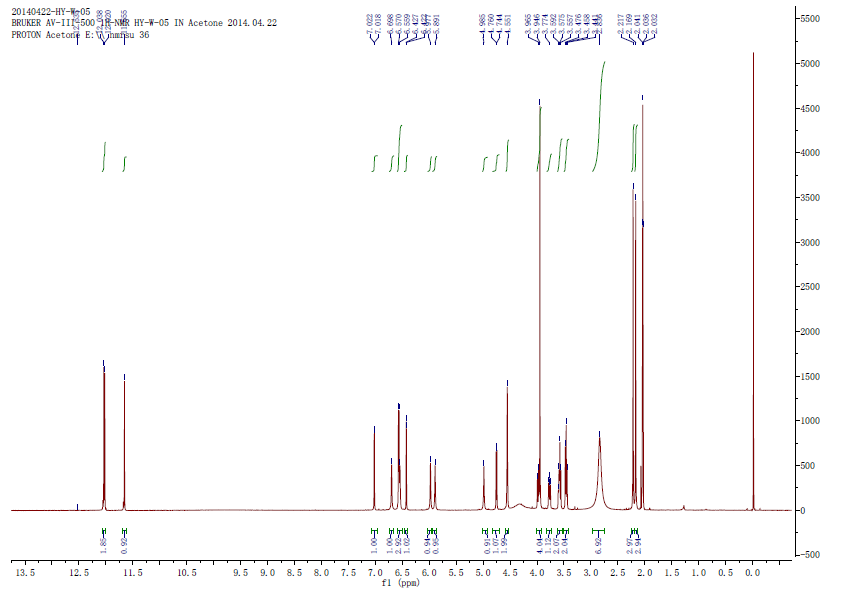


Polygonumnolide A3. ^13^C NMR (150 MHz, CD_3_COCD_3_) spectrum of the new compound **3**


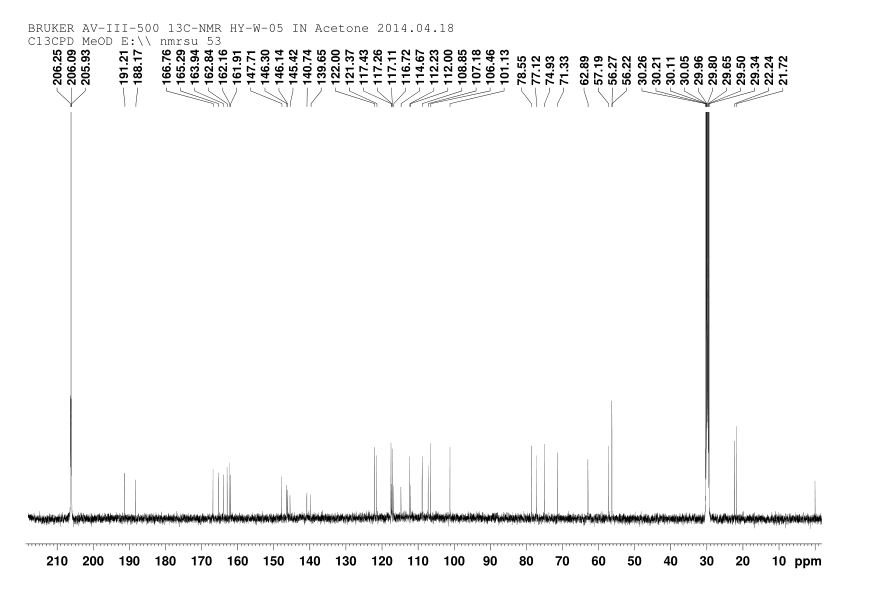


Polygonumnolide A3. DEPT spectrum of the new compound **3**


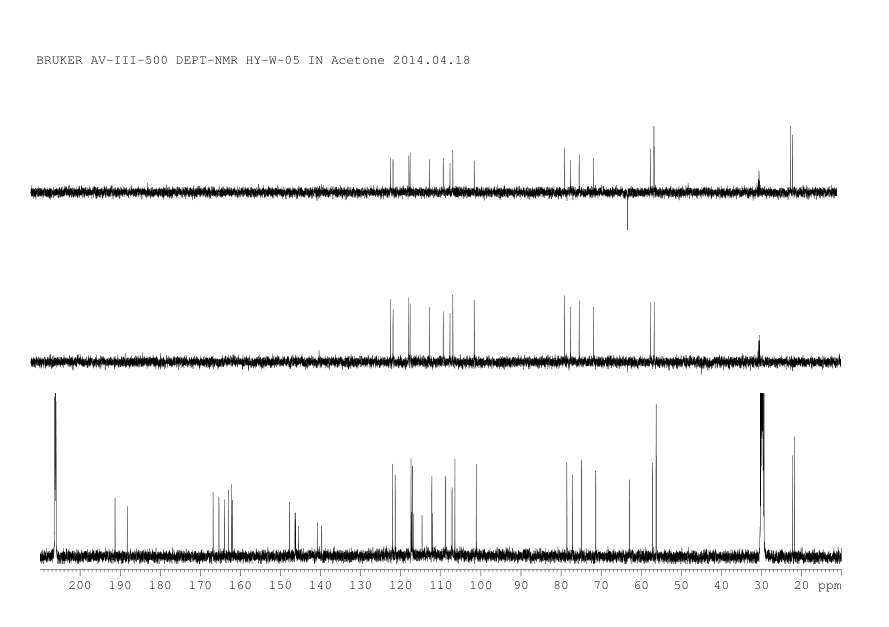


Polygonumnolide A3. ^1^H-^1^H COSY spectrum of the new compound **3**


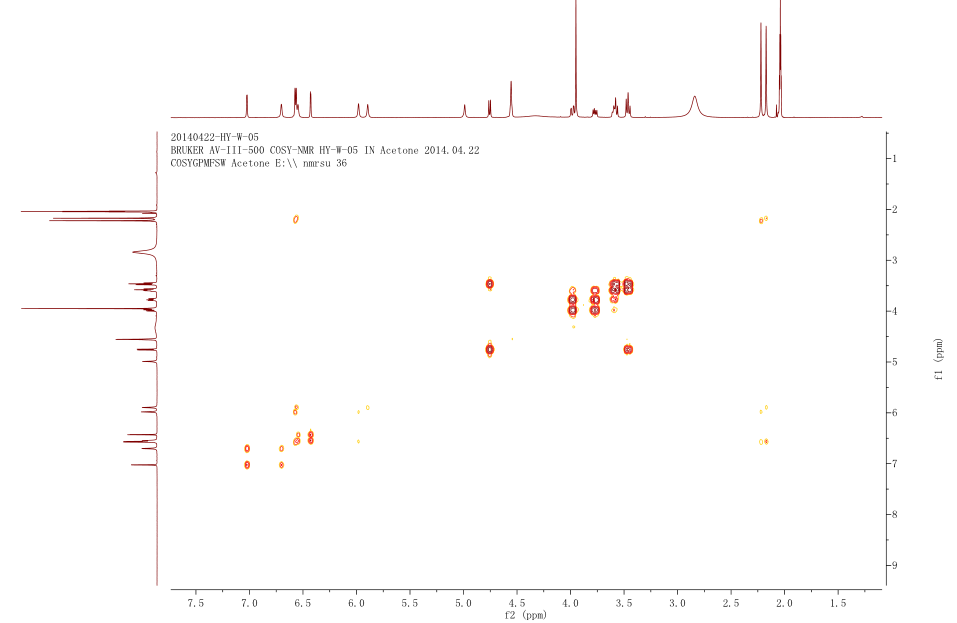


Polygonumnolide A3. HSQC spectrum of the new compound **3**


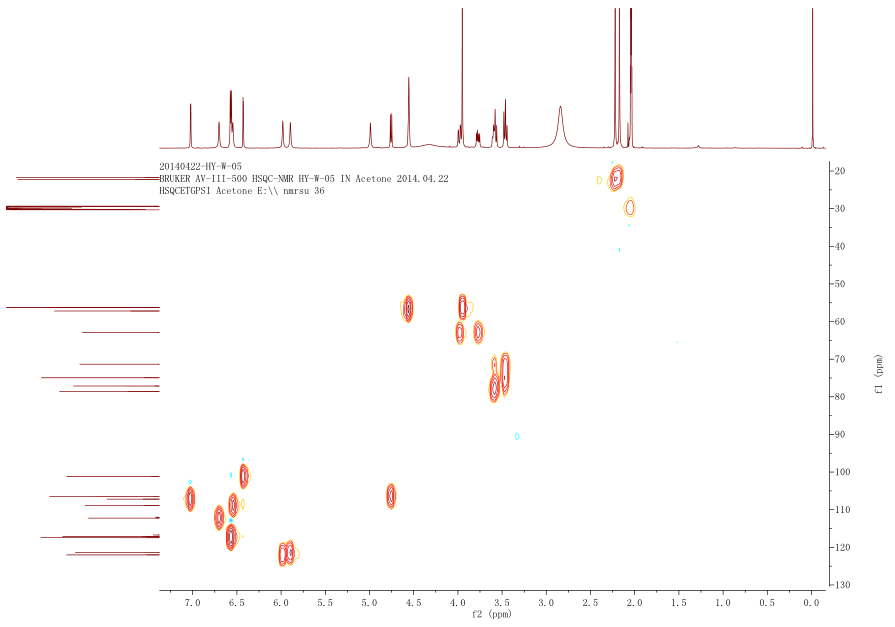


Polygonumnolide A3. HMBC spectrum of the new compound **3**


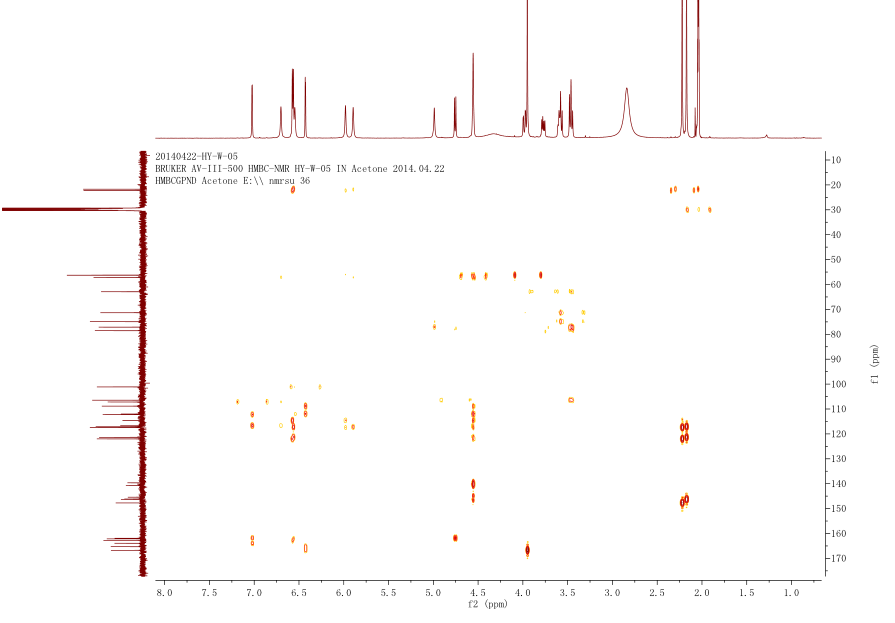


Polygonumnolide A4. IR spectrum of the new compound **4**


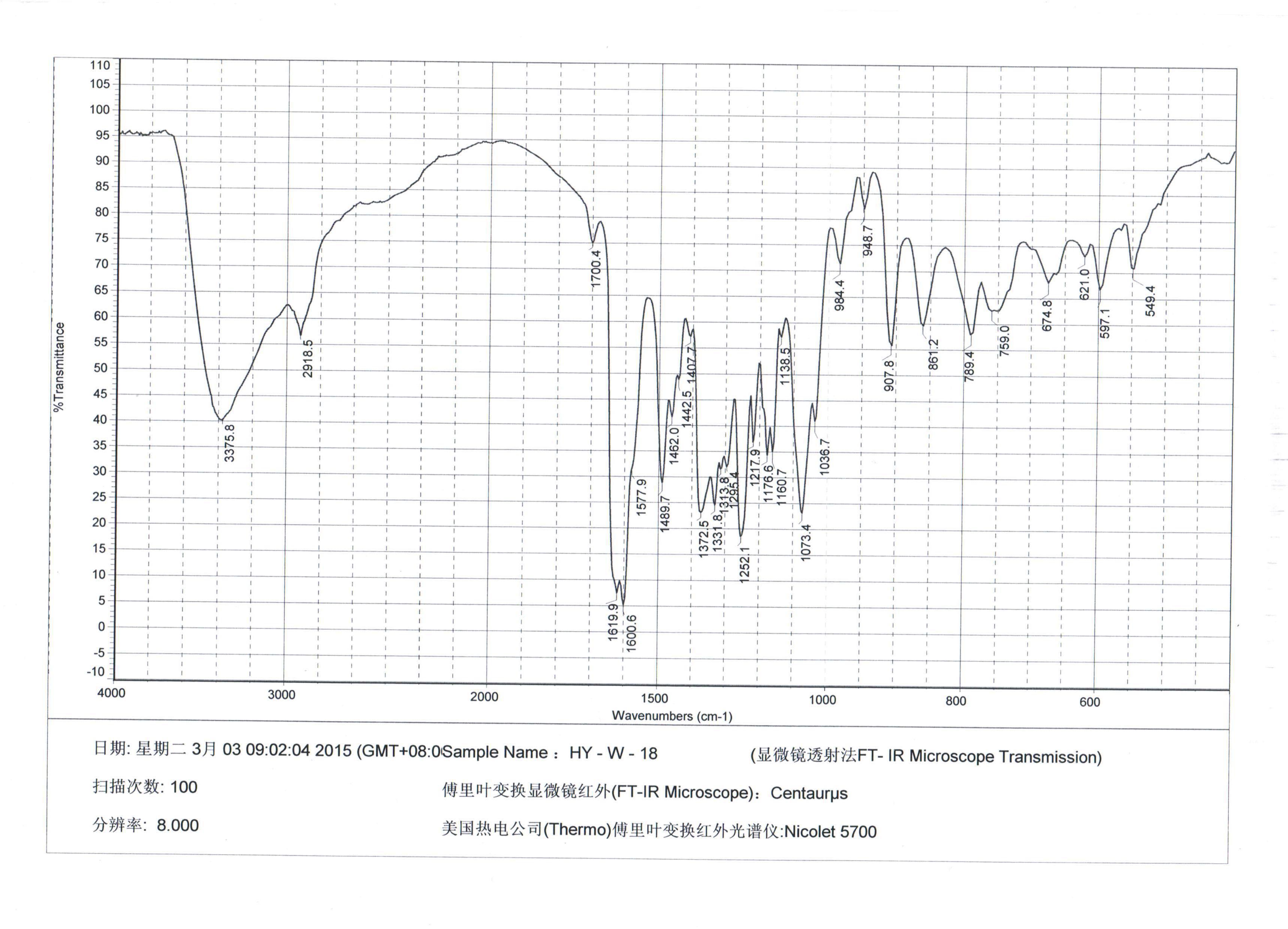


Polygonumnolide A4. HRESIMS spectrum of the new compound **4**


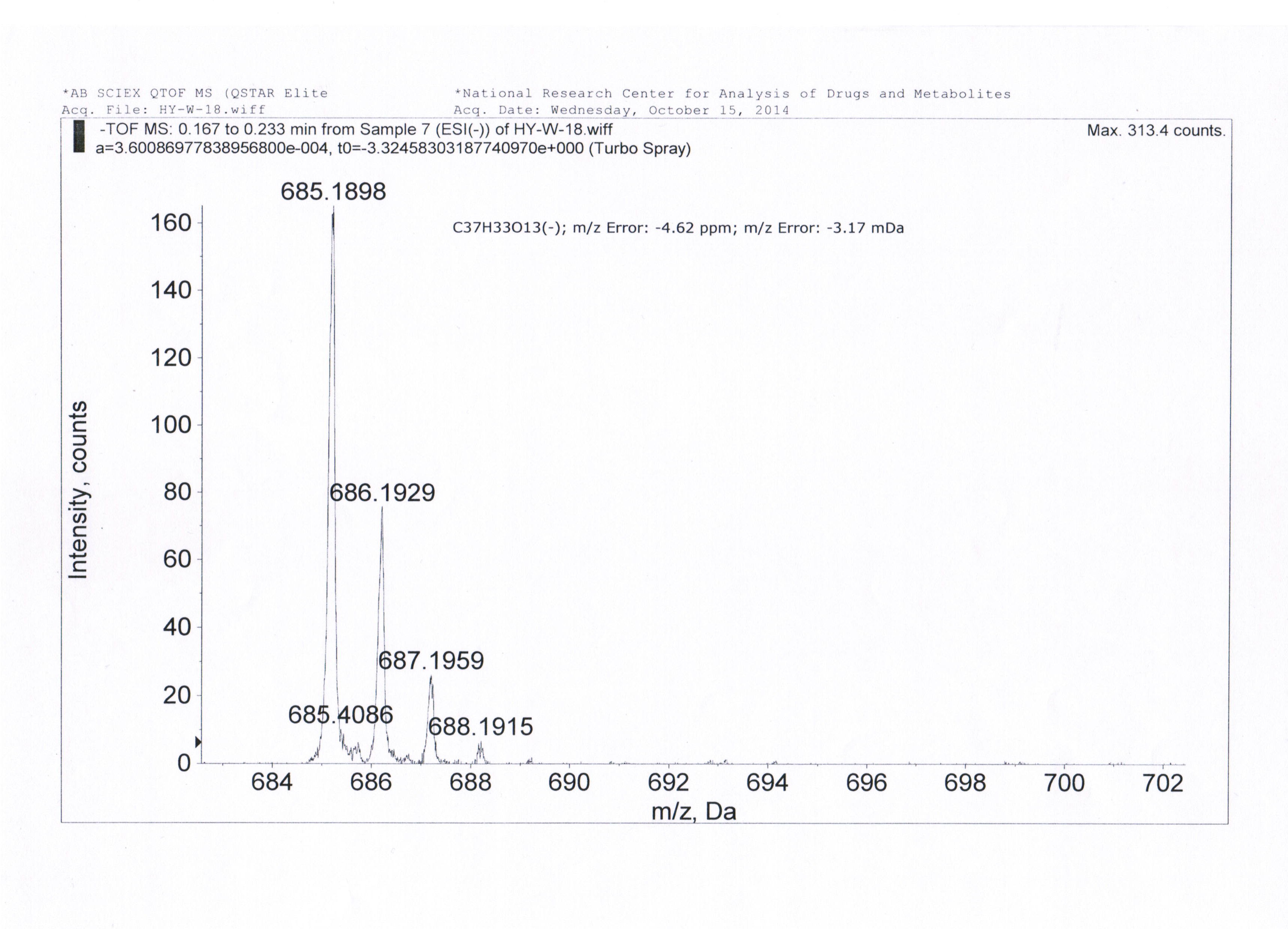


Polygonumnolide A4. ^1^H NMR (600 MHz, CD_3_OD) spectrum of the new compound **4**


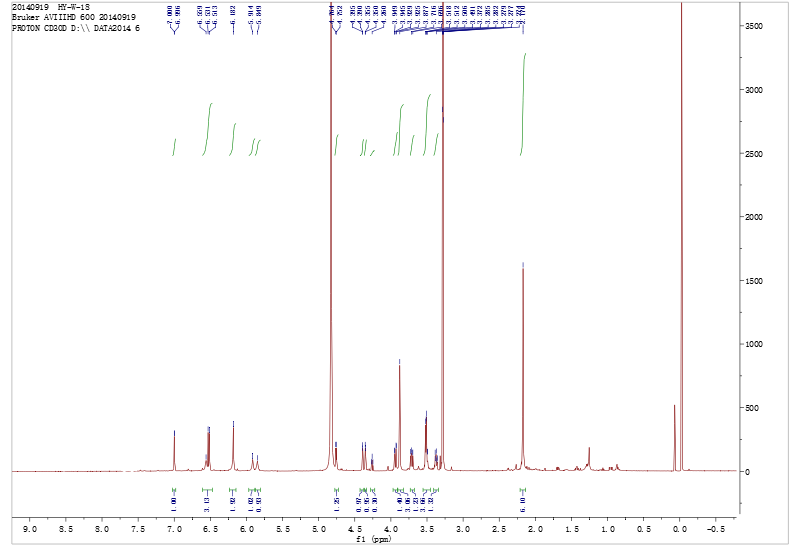


Polygonumnolide A4. ^13^C NMR (150 MHz, CD_3_OD) spectrum of the new compound **4**


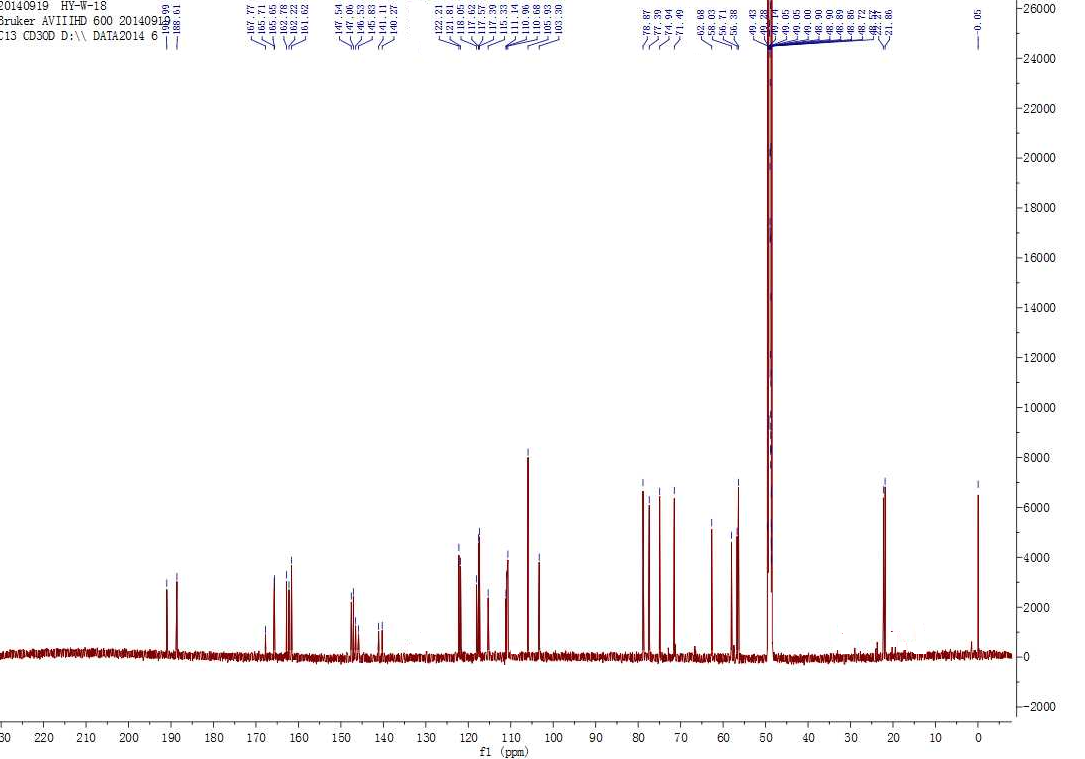


Polygonumnolide A4. DEPT spectrum of the new compound **4**


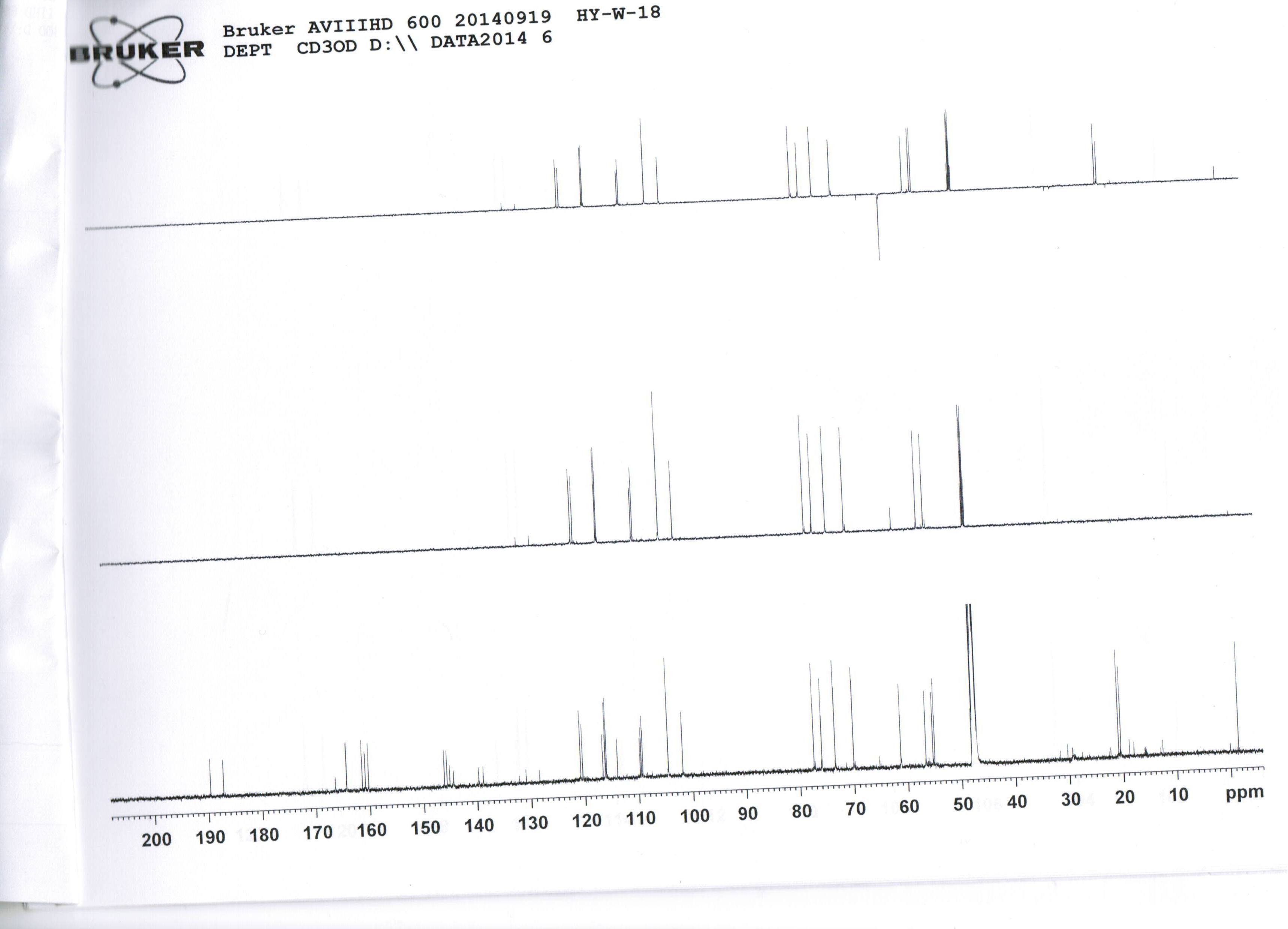


PolygonumnolideA4. ^1^H-^1^H COSY spectrum of the new compound **4**


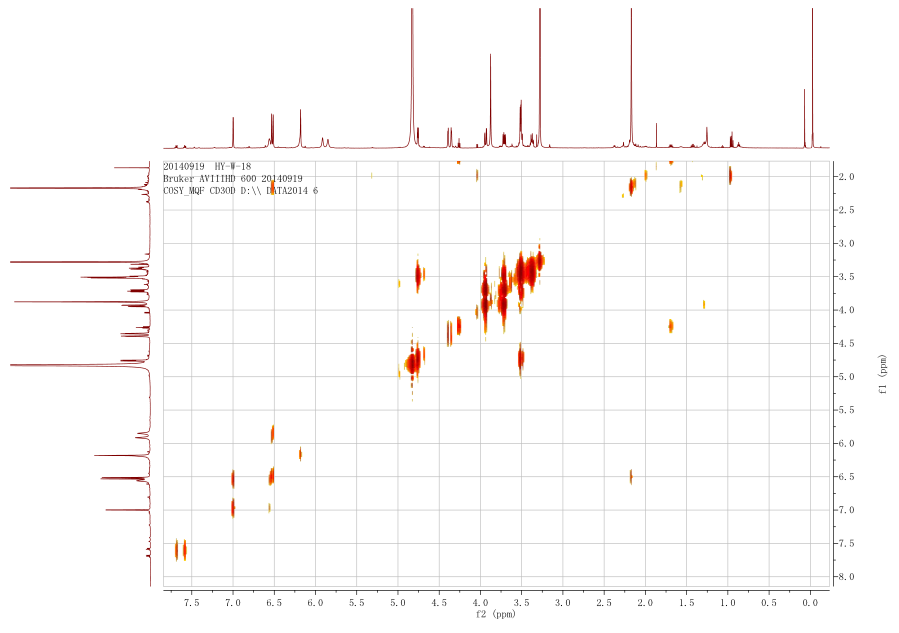


Polygonumnolide A4. HSQC spectrum of the new compound **4**


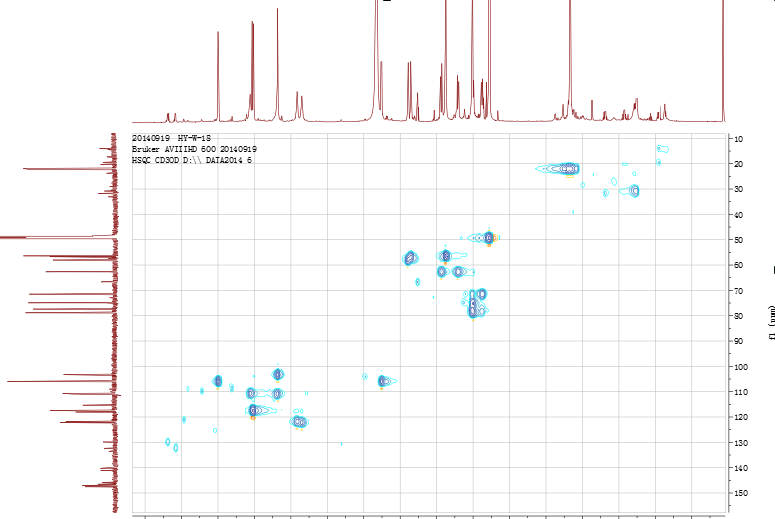


Polygonumnolide A4. HMBC spectrum of the new compound **4**


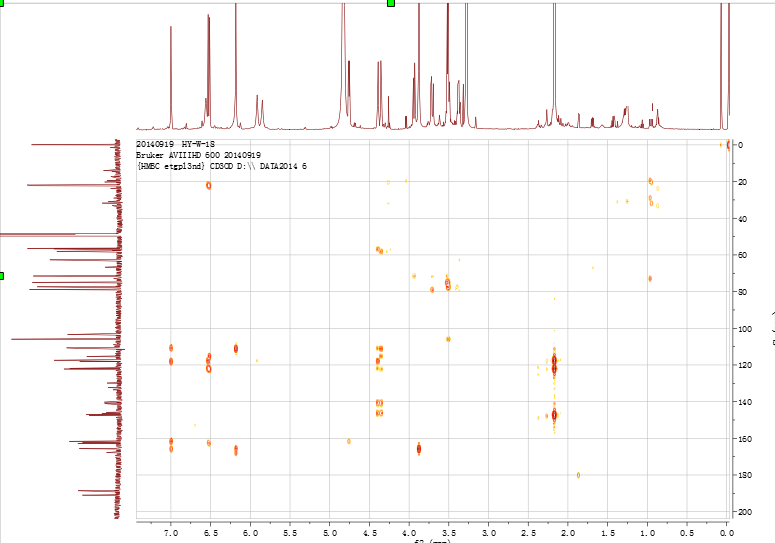


Polygonumnolide A4. ROESY spectrum of the new compound **4**

**
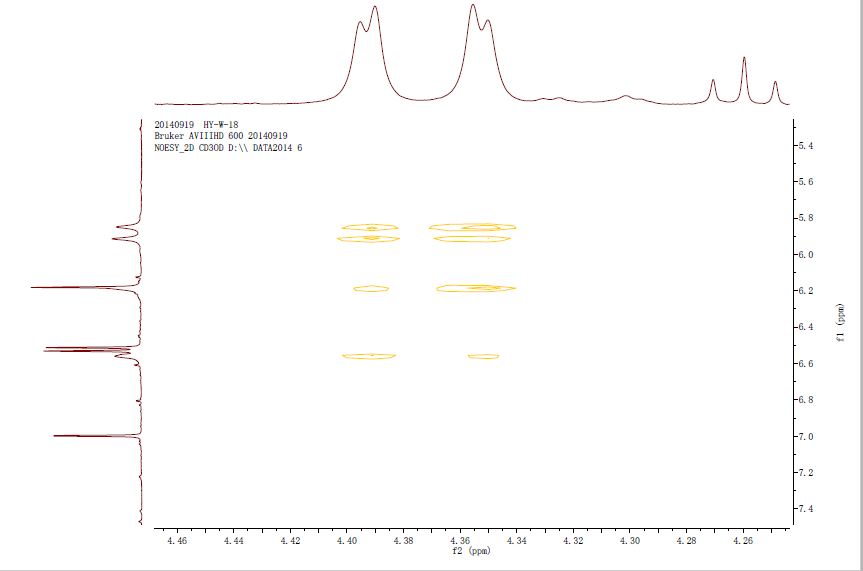
**

Polygonumnolide B1. IR spectrum of the new compound **5**


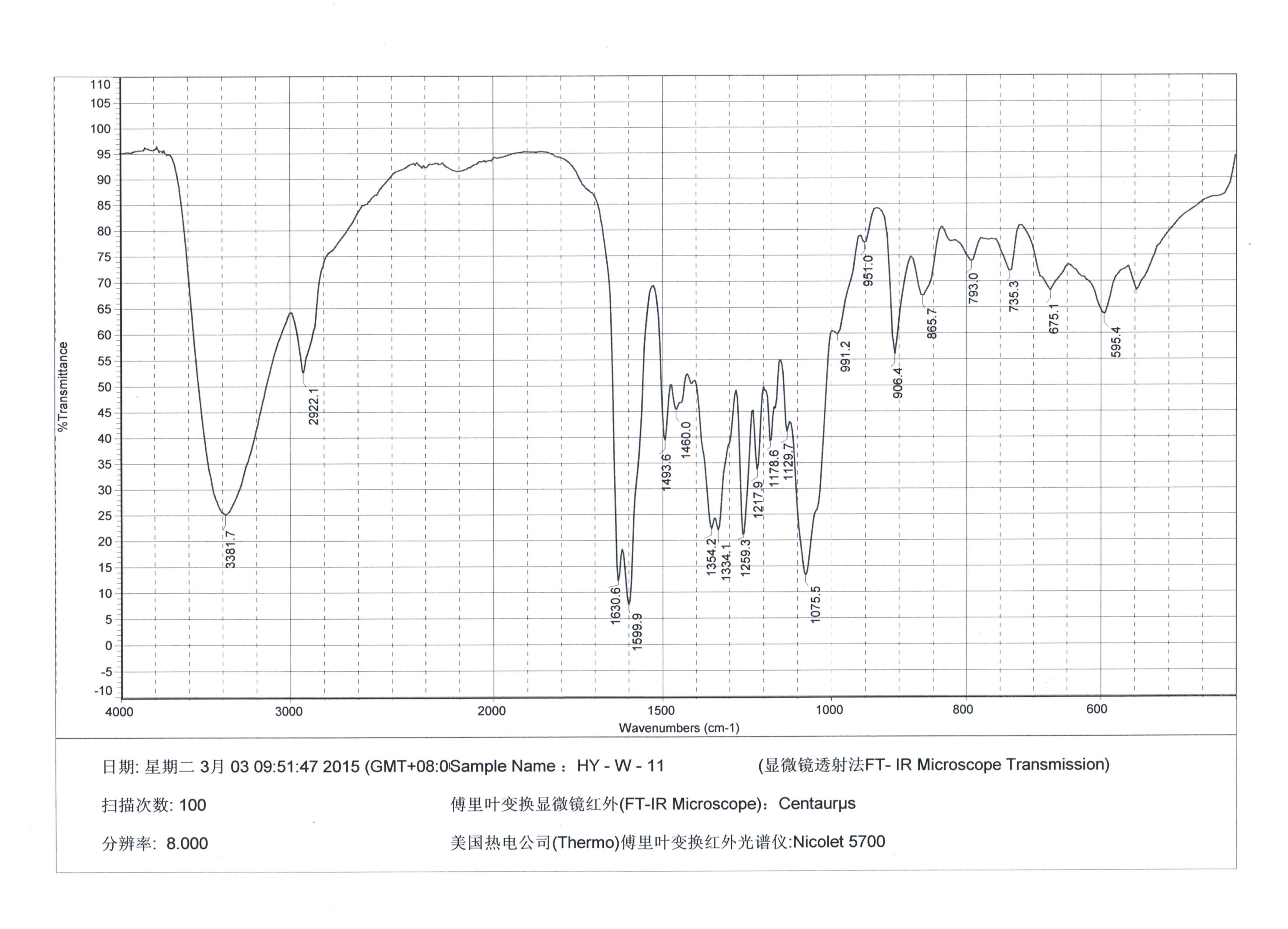


Polygonumnolide B1. HRESIMS spectrum of the new compound **5**

Polygonumnolide B1. ^1^H NMR (600 MHz, CD_3_OD) spectrum of the new compound **5**


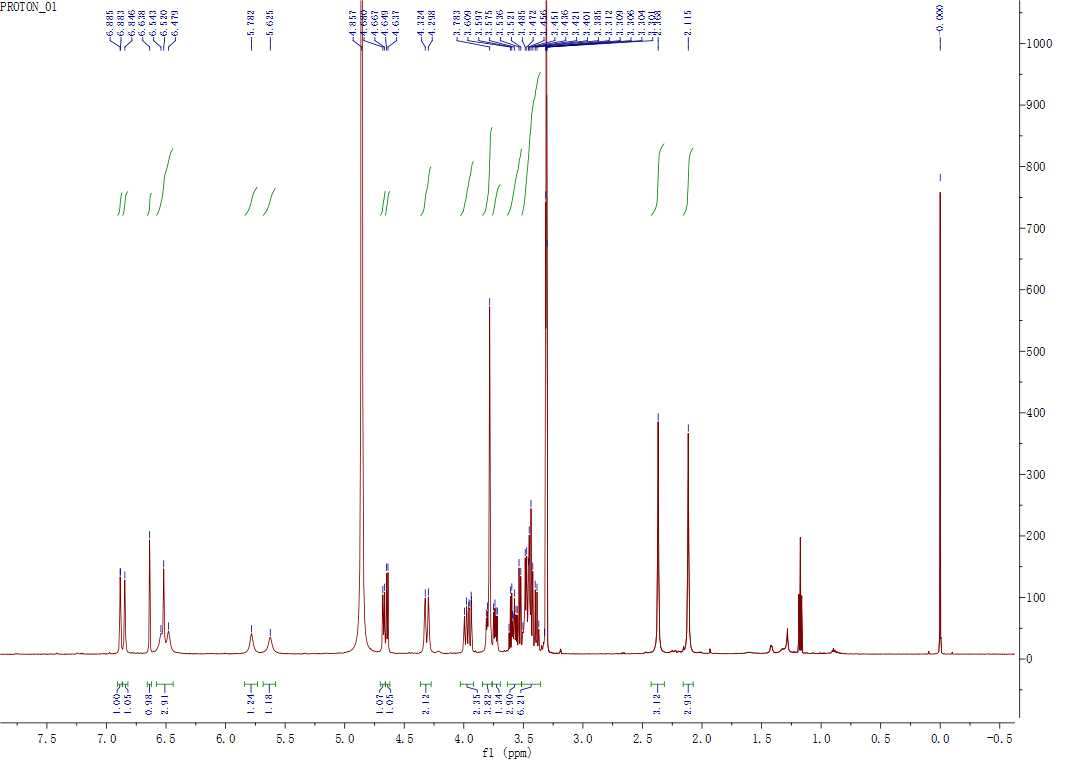


Polygonumnolide B1. ^13^C NMR (150 MHz, CD_3_OD) spectrum of the new compound **5**


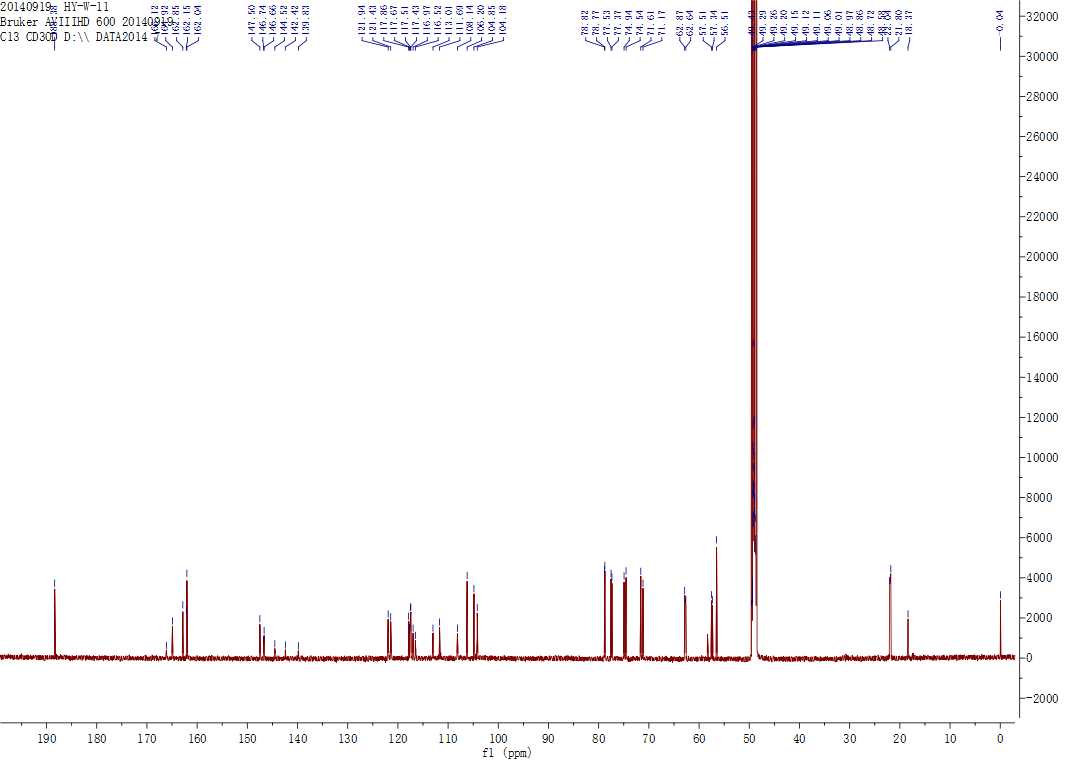


Polygonumnolide B1. DEPT spectrum of the new compound **5**


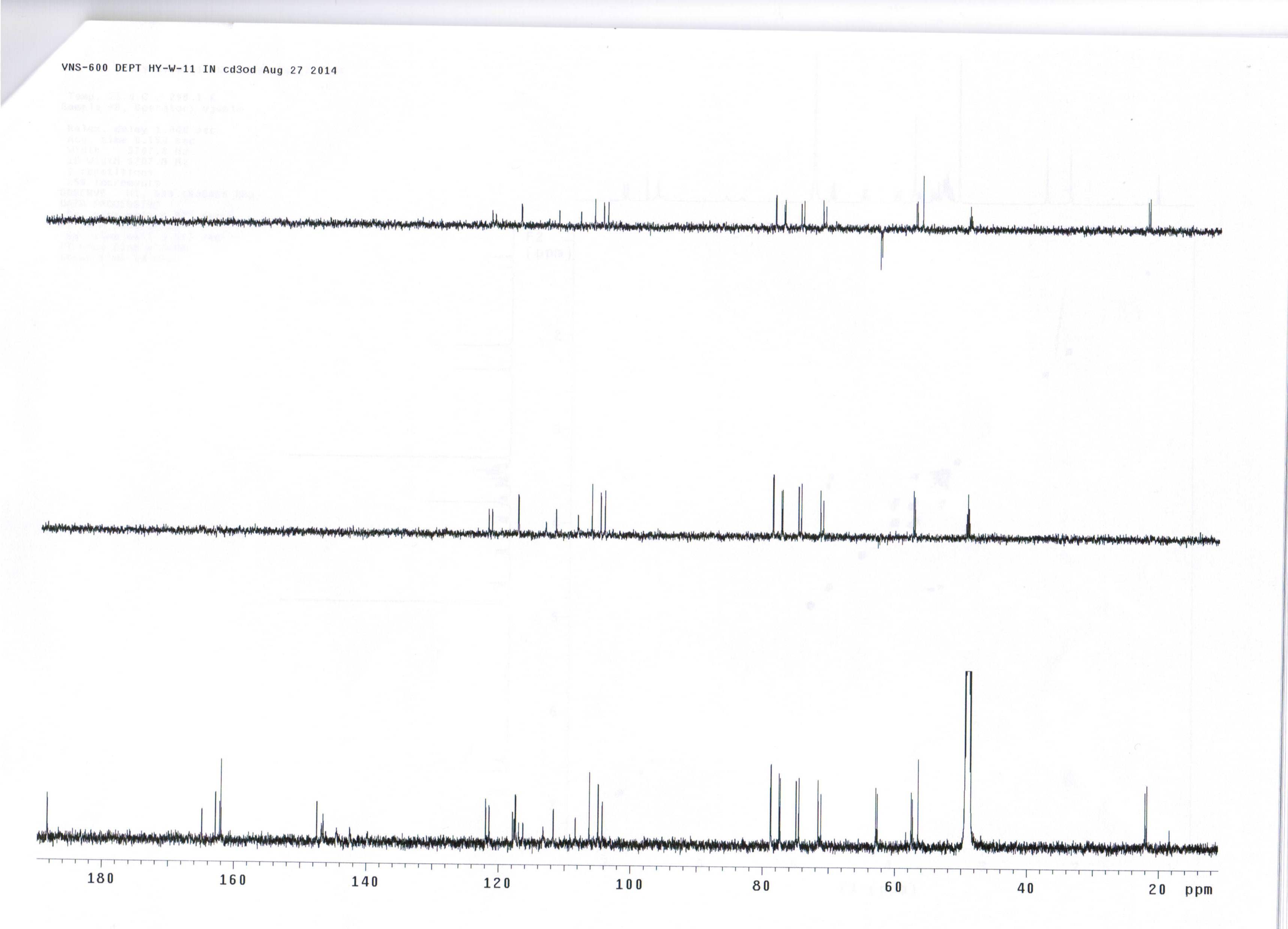


Polygonumnolide B1. ^1^H-^1^H COSY spectrum of the new compound **5**


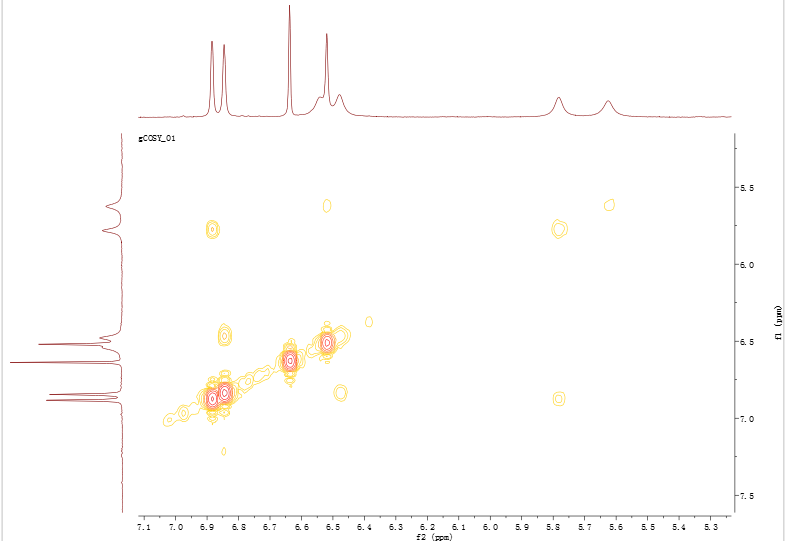


Polygonumnolide B1. HSQC spectrum of the new compound **5**


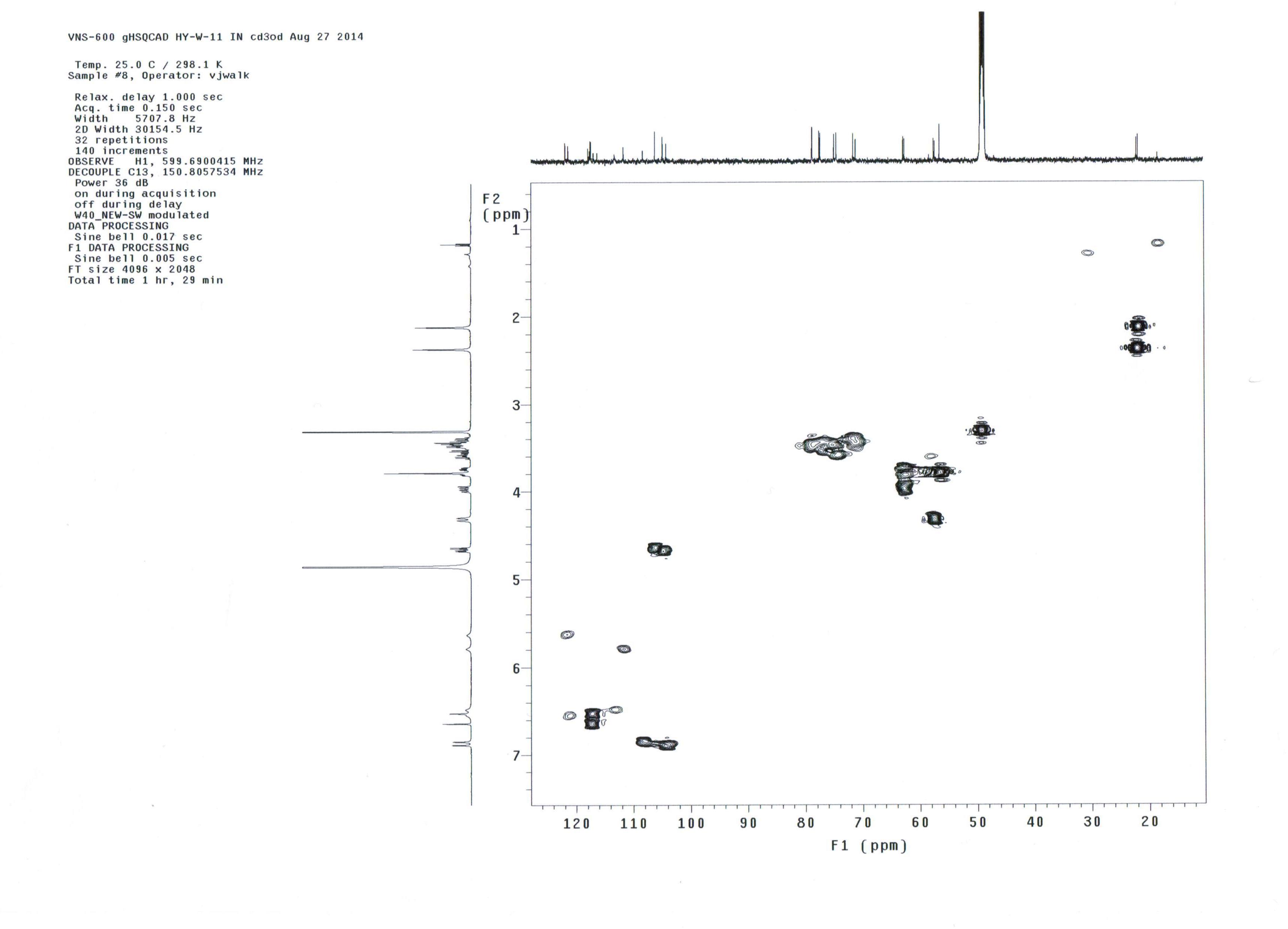


Polygonumnolide B1. HMBC spectrum of the new compound **5**


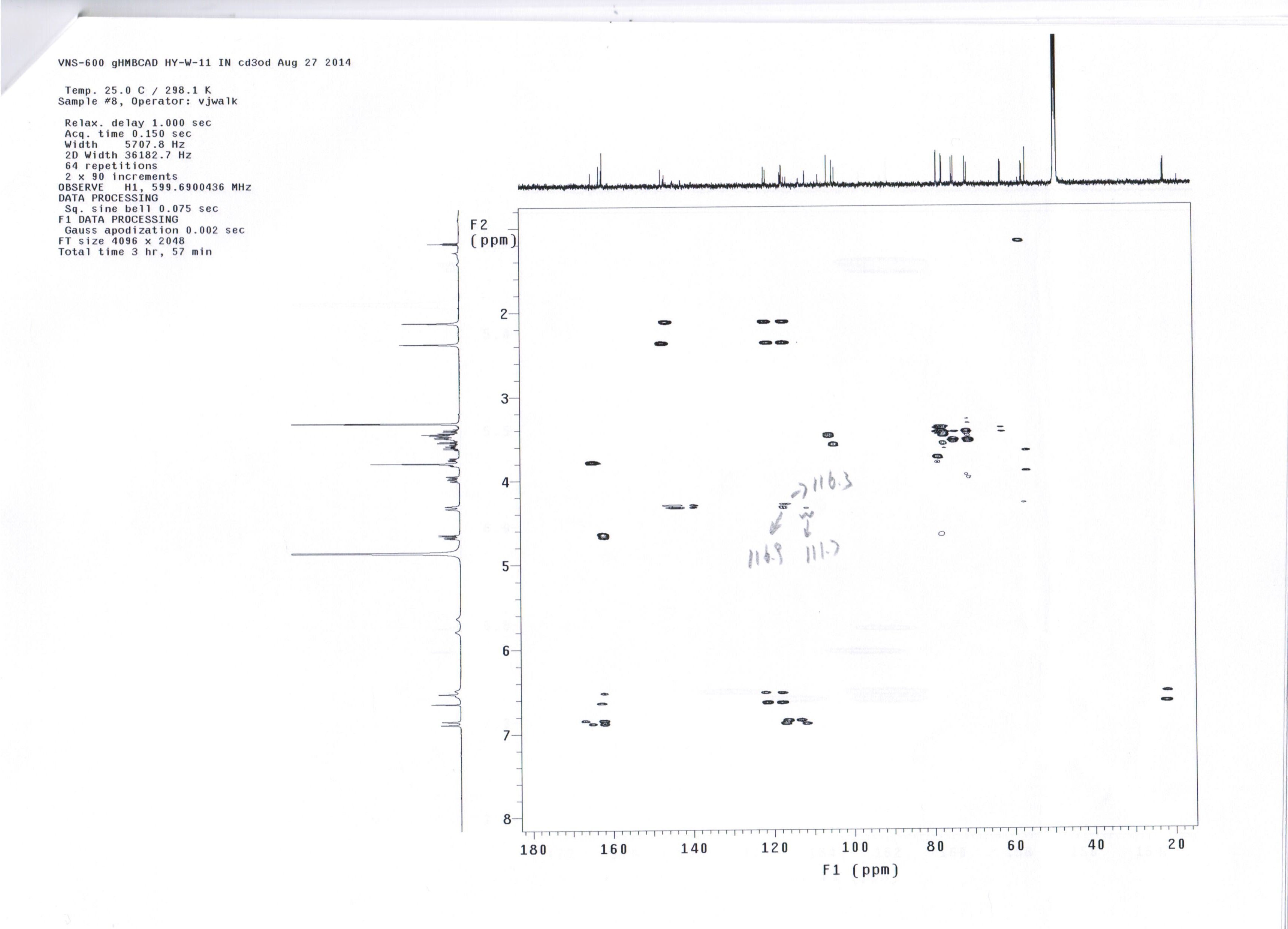


Polygonumnolide B1. ROESY spectrum of the new compound **5**

**
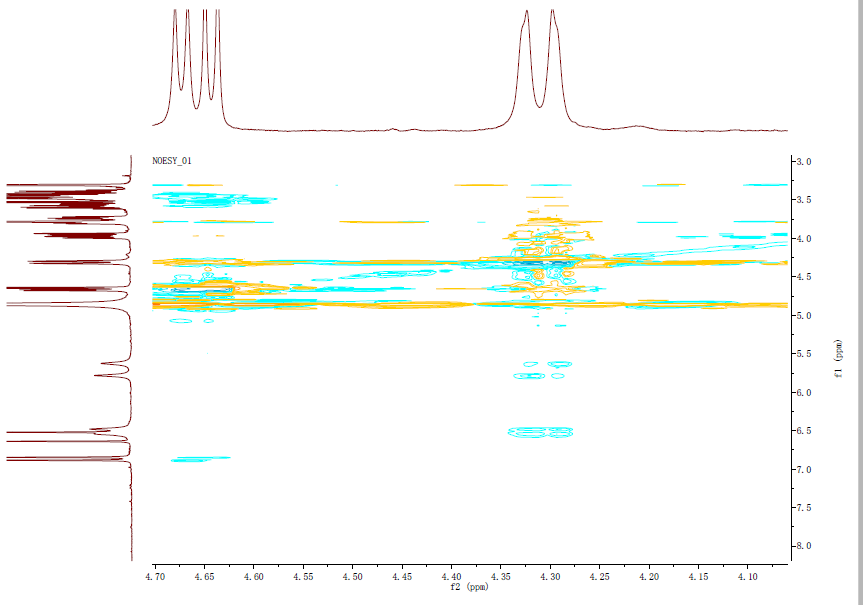
**

Polygonumnolide B2. IR spectrum of the new compound **6**


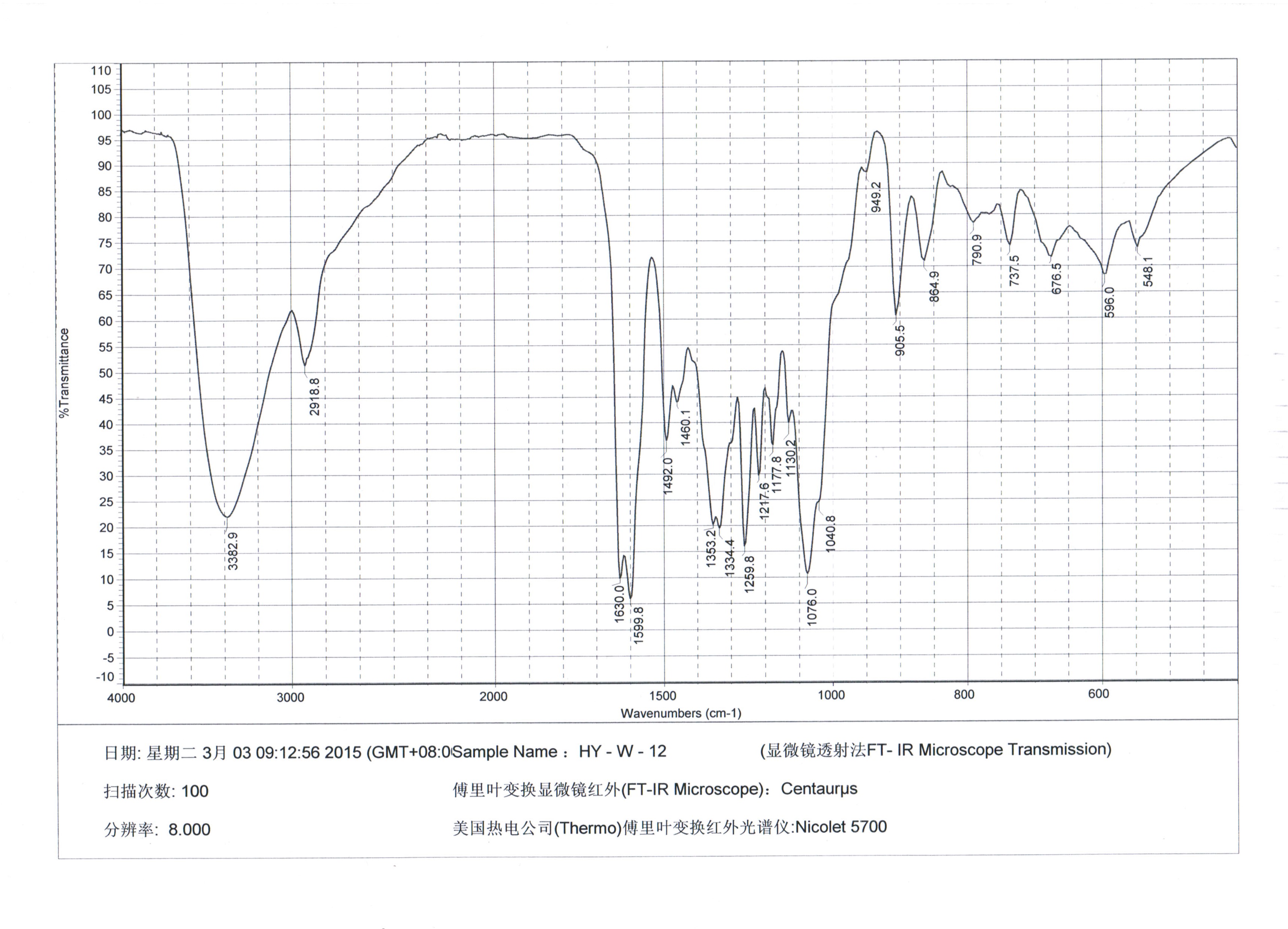


Polygonumnolide B2. HRESIMS spectrum of the new compound **6**

Polygonumnolide B2. ^1^H NMR (600 MHz, CD_3_OD) spectrum of the new compound **6**


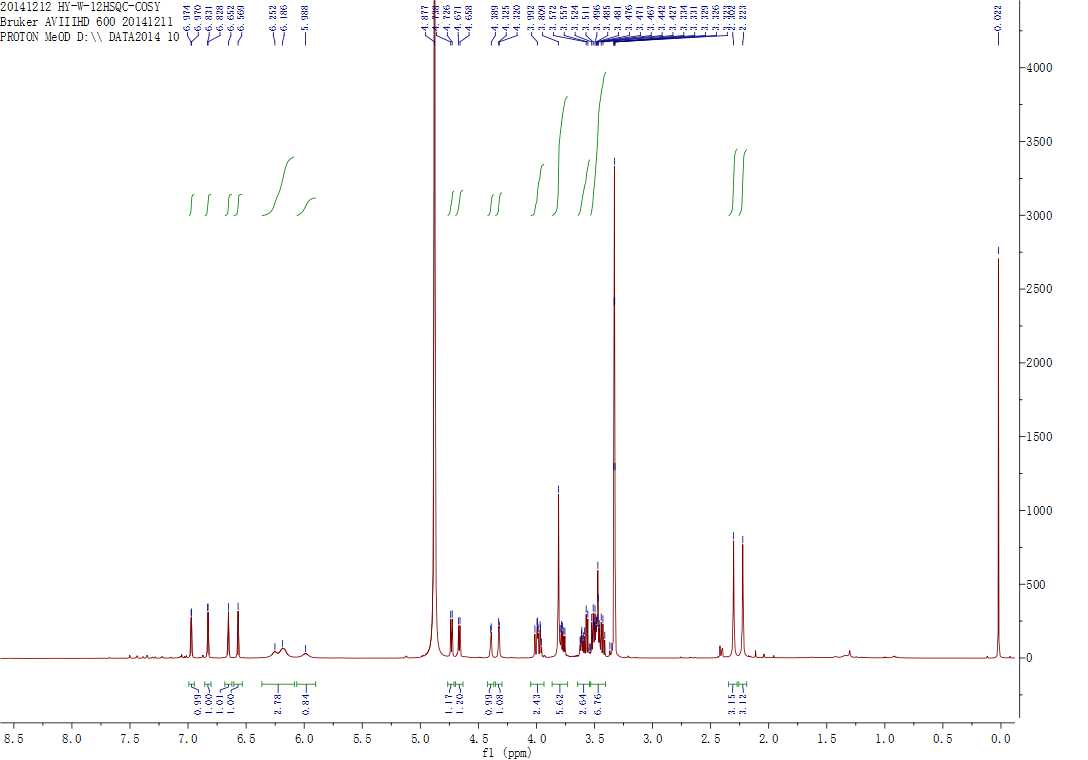


Polygonumnolide B2. ^13^C NMR (150 MHz, CD_3_OD) spectrum of the new compound **6**


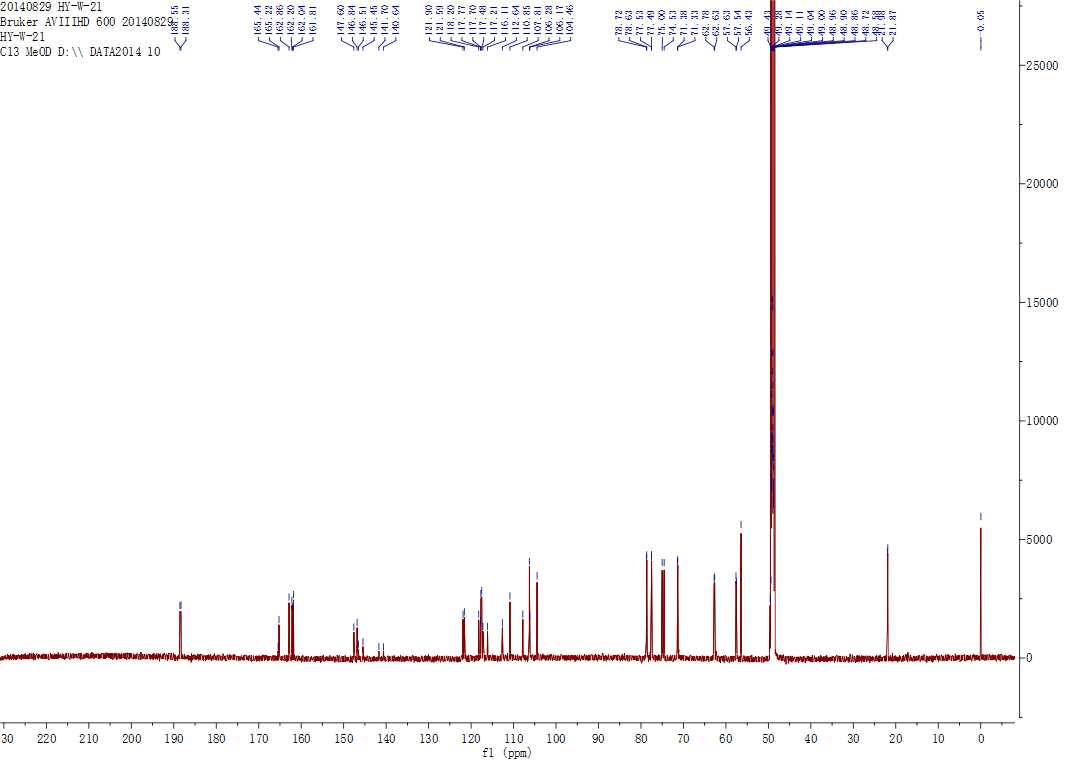


Polygonumnolide B2. DEPT spectrum of the new compound **6**


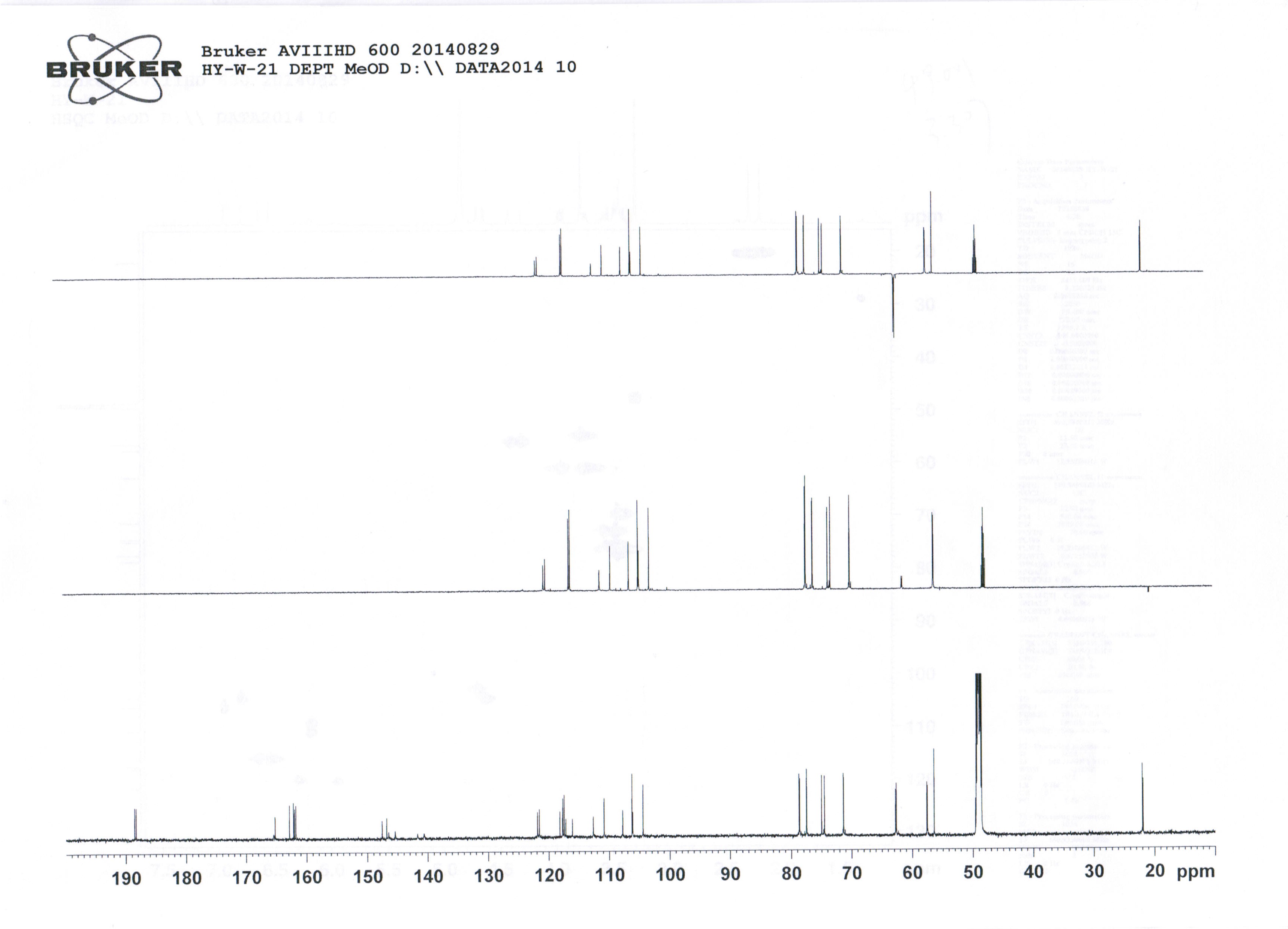


Polygonumnolide B2. ^1^H-^1^H COSY spectrum of the new compound **6**


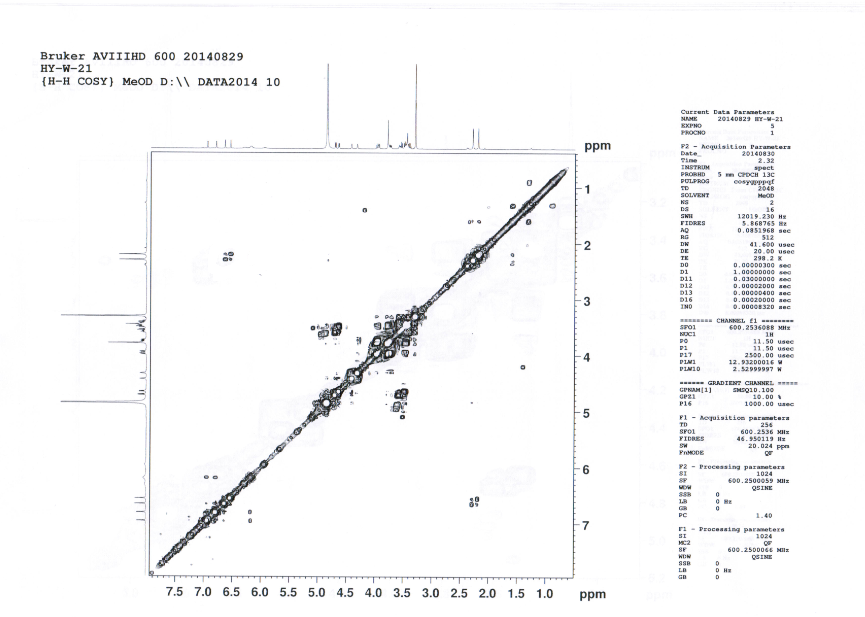


Polygonumnolide B2. HSQC spectrum of the new compound **6**


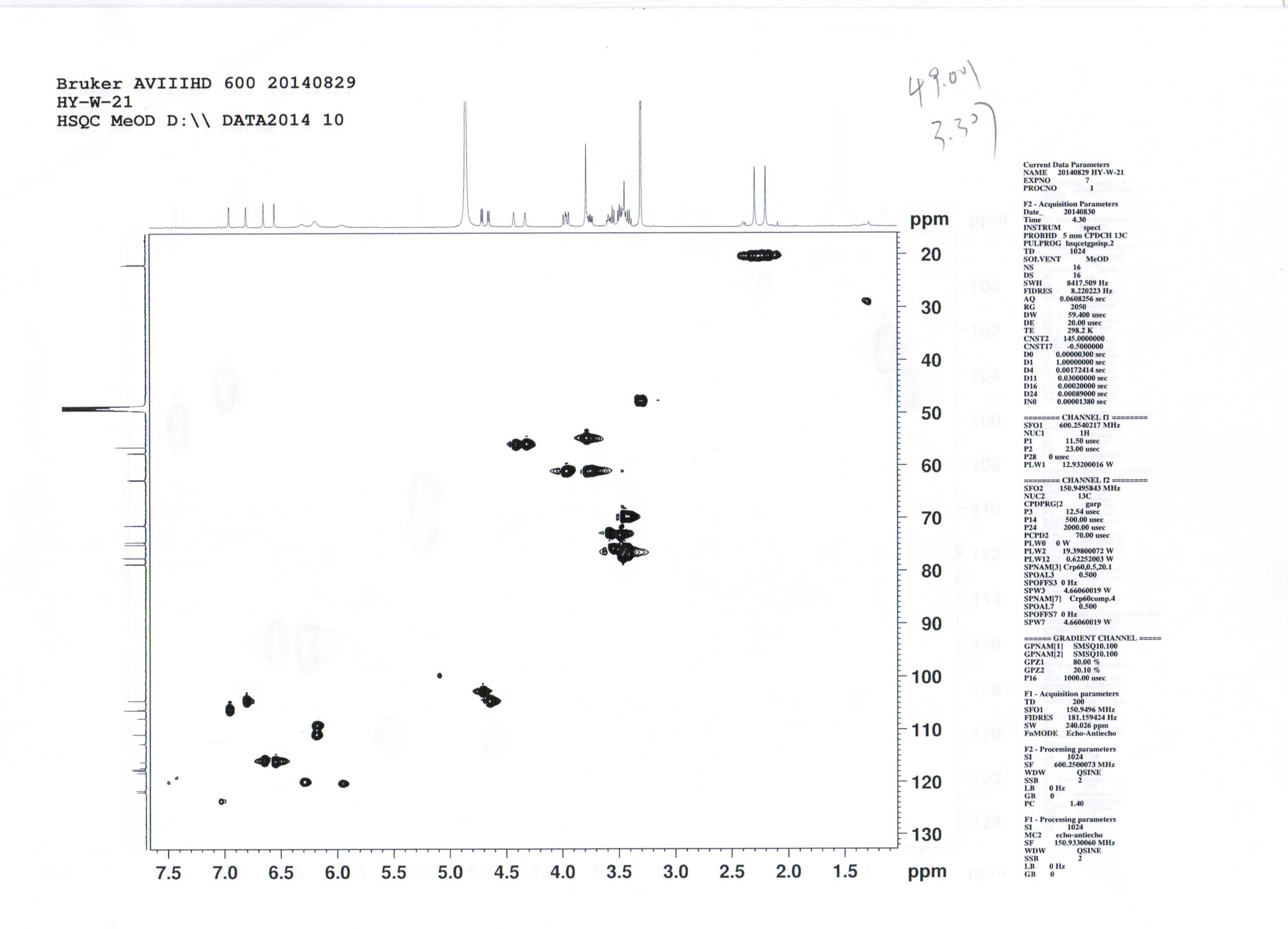


Polygonumnolide B2. HMBC spectrum of the new compound **6**


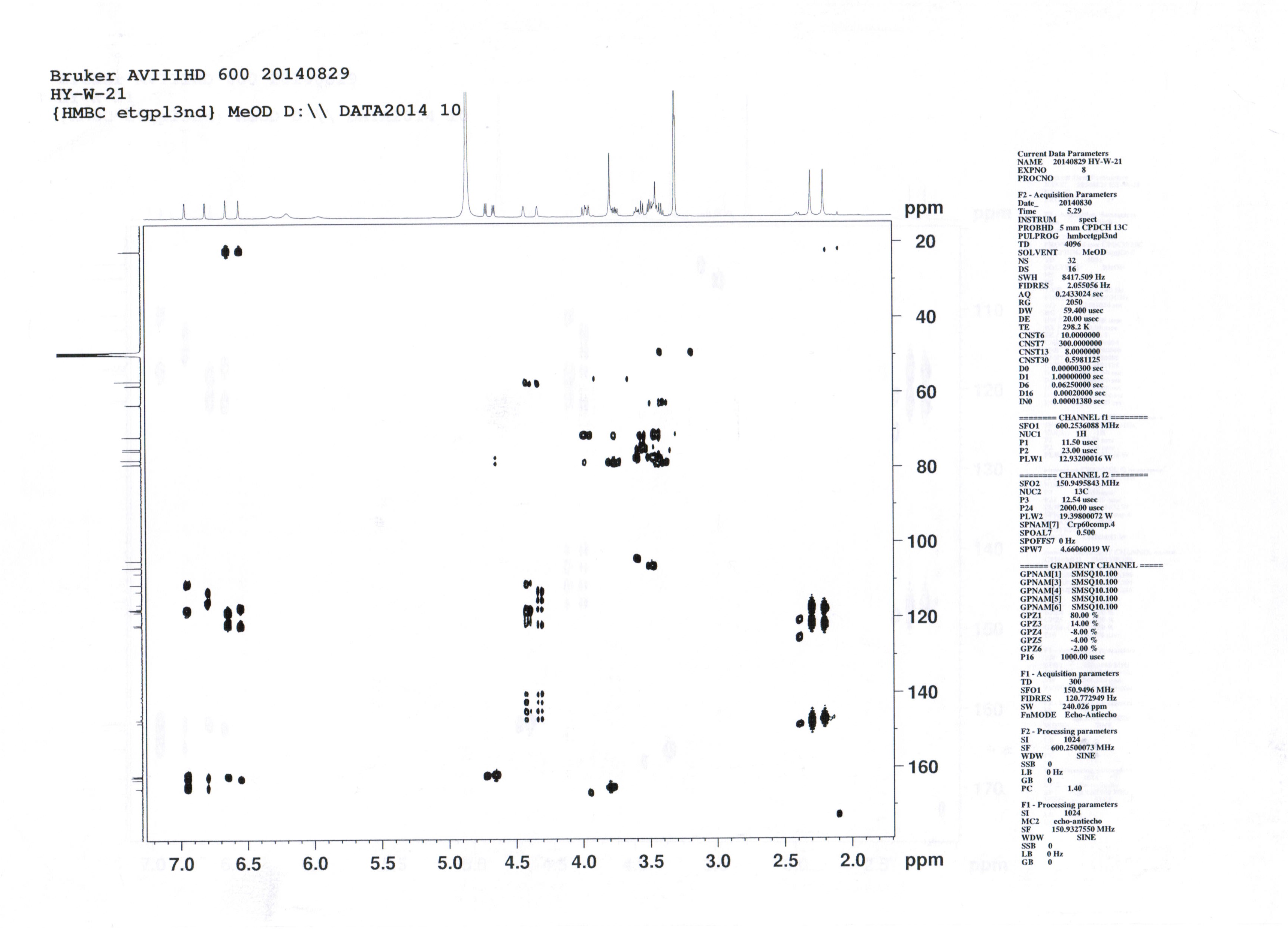


Polygonumnolide B2. ROESY spectrum of the new compound **6**

**
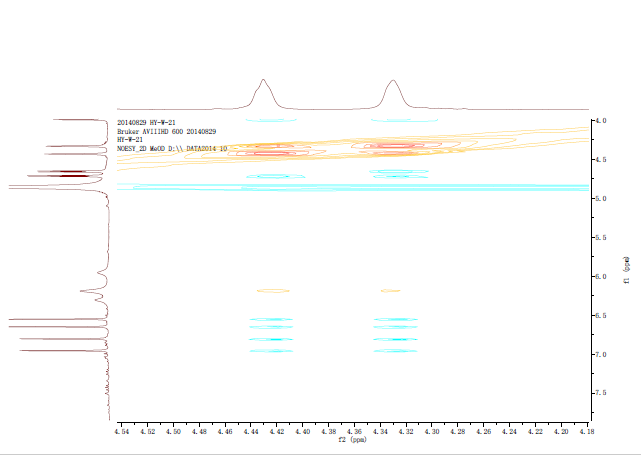
**

Polygonumnolide B3. IR spectrum of the new compound **7**


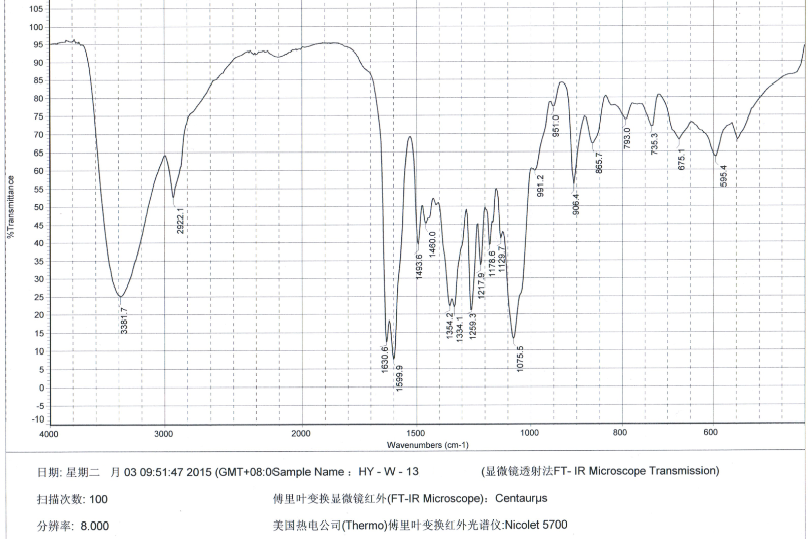


Polygonumnolide B3. HRESIMS spectrum of the new compound **7**

Polygonumnolide B3. ^1^H NMR (600 MHz, CD_3_OD) spectrum of the new compound **7**


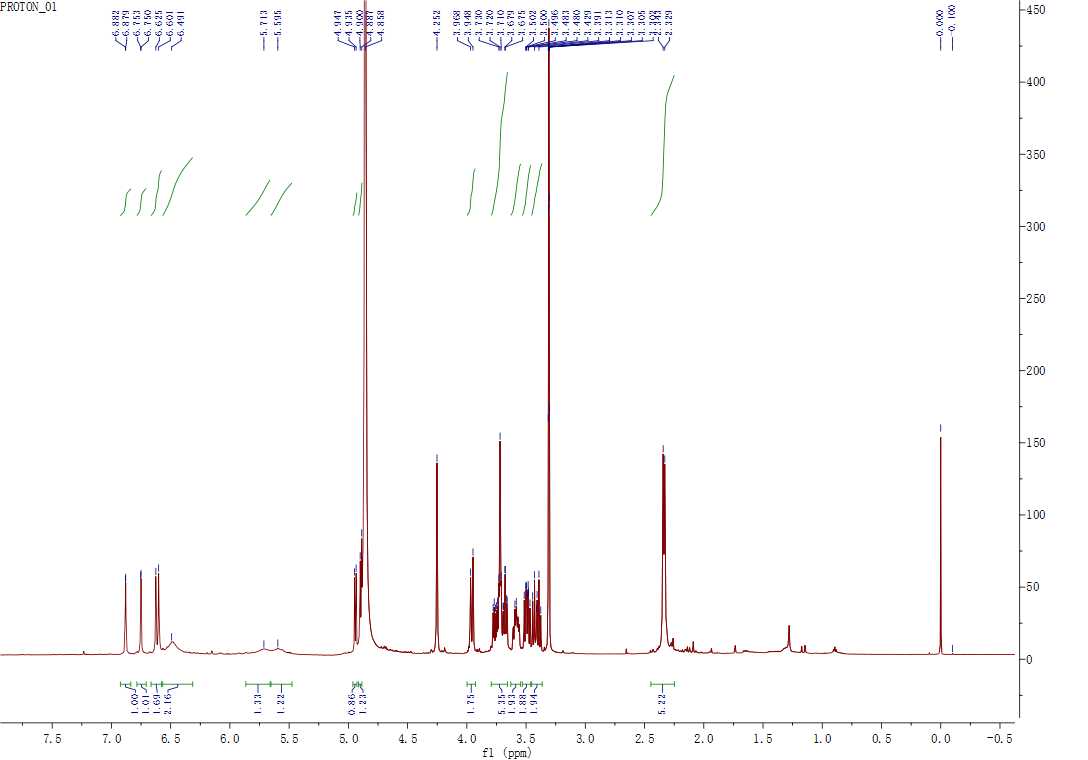


Polygonumnolide B3. ^13^C NMR (150 MHz, CD_3_OD) spectrum of the new compound **7**


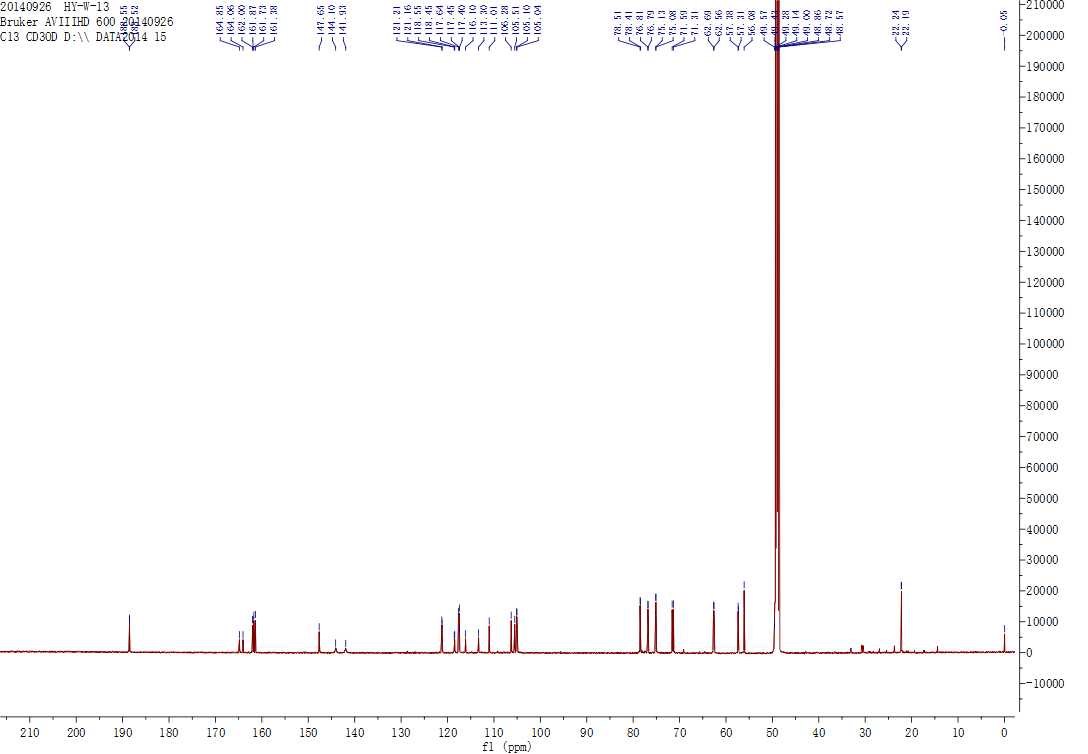


Polygonumnolide B3. DEPT spectrum of the new compound **7**


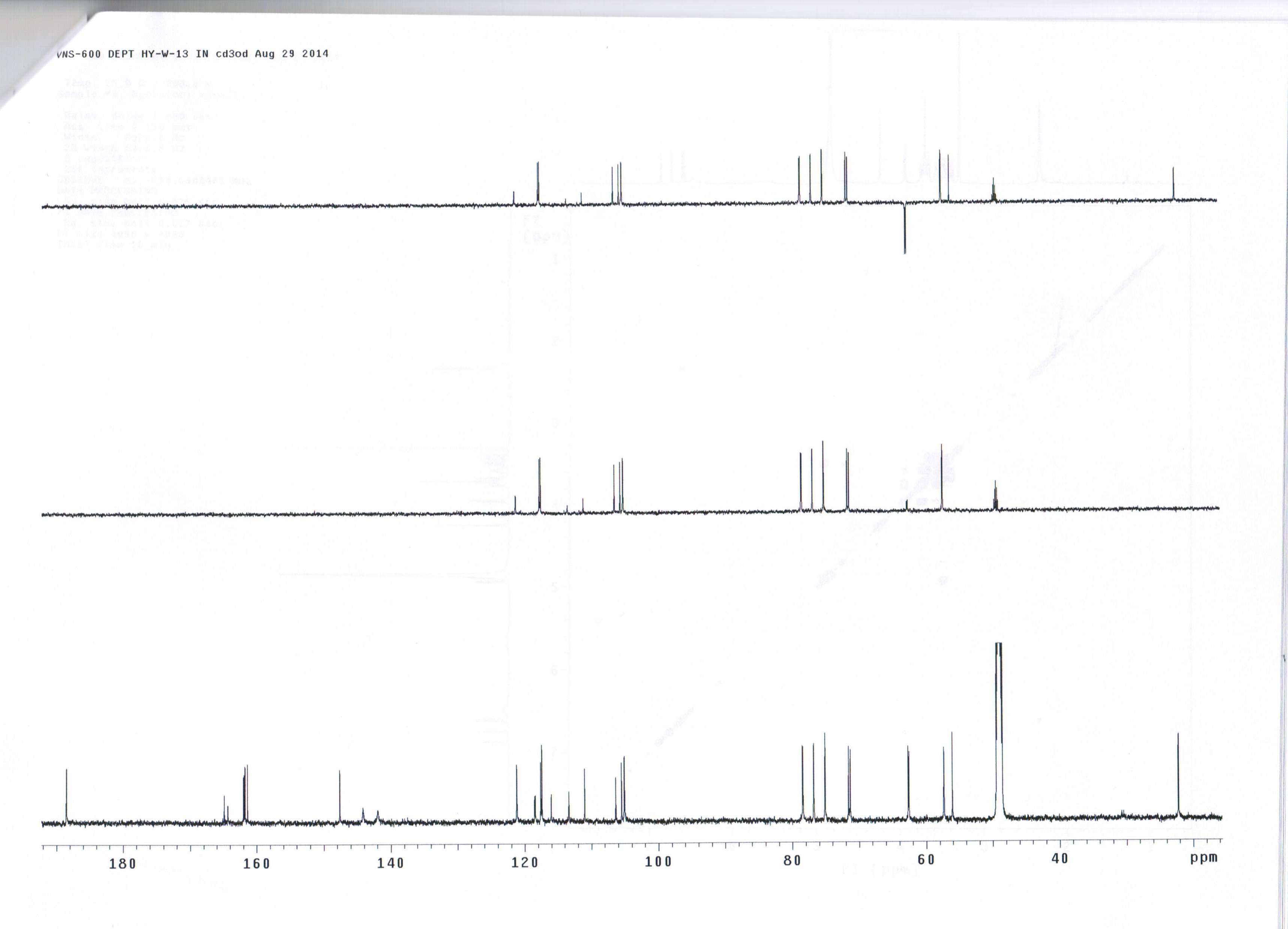


Polygonumnolide B3. ^1^H-^1^H COSY spectrum of the new compound **7**


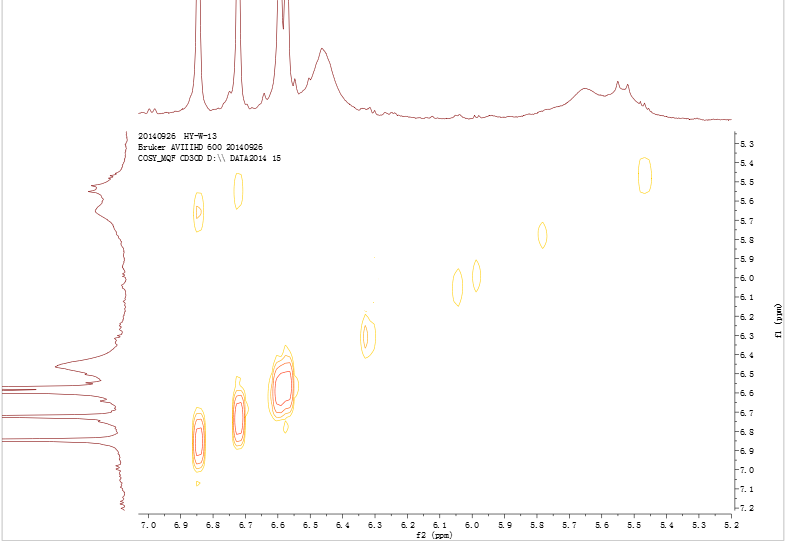


Polygonumnolide B3. HSQC spectrum of the new compound **7**


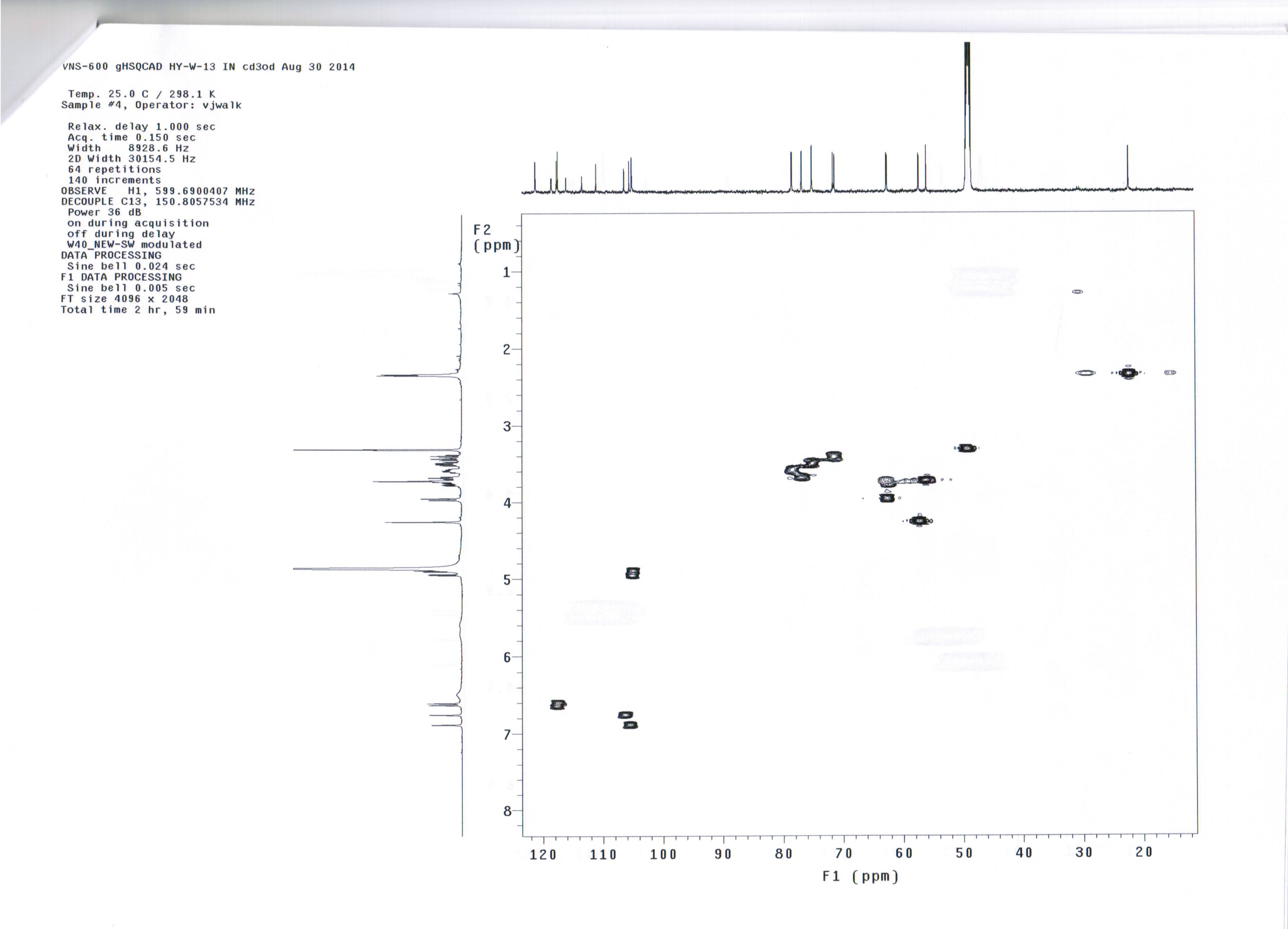


Polygonumnolide B3. HMBC spectrum of the new compound **7**


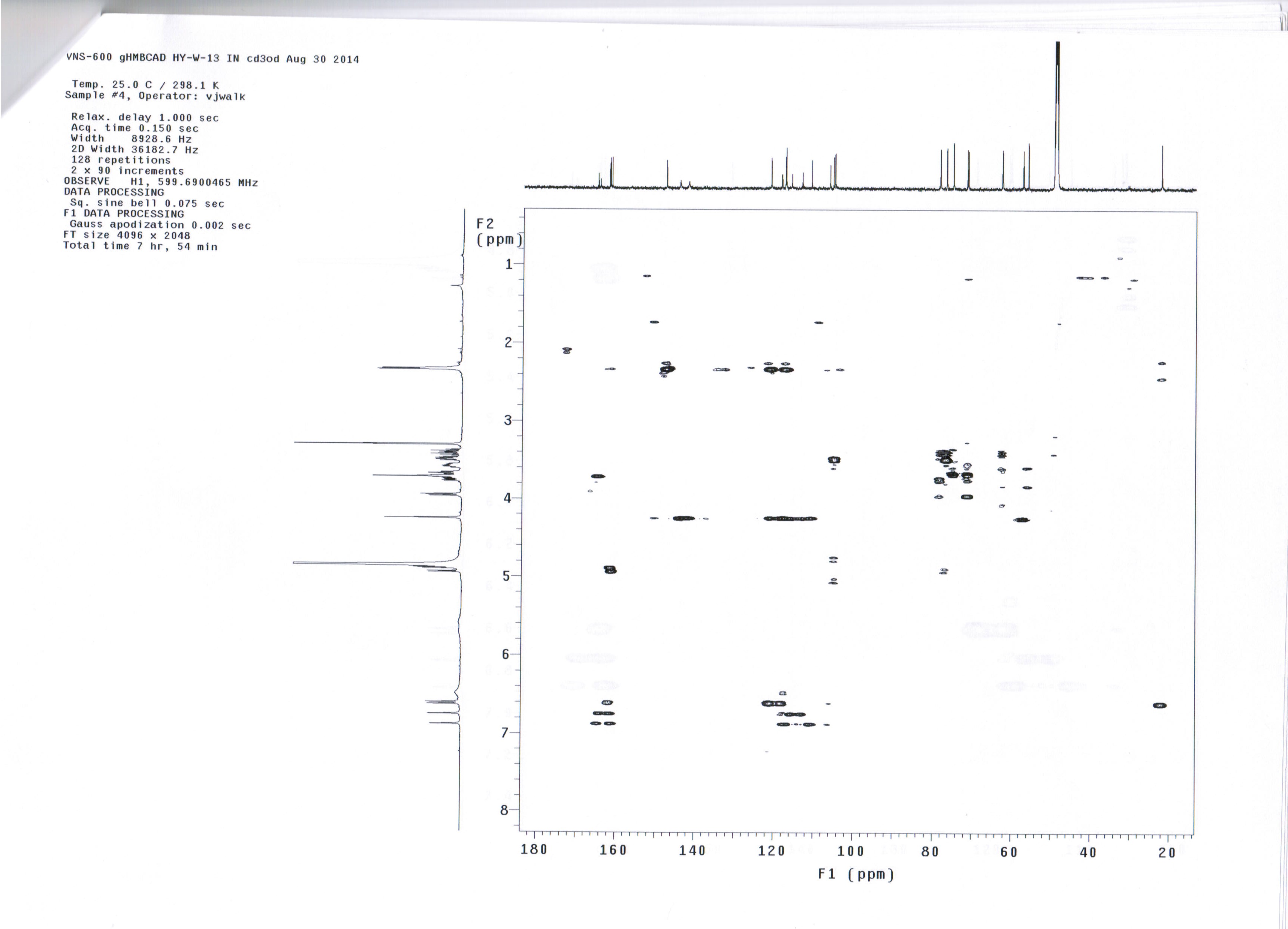

Supplement: Supplementary file 1 — Spectroscopic data for the new compounds (1–7) including 1H, 13C, and 2D NMR, IR and ESI-MS/MS data are provided in the supporting information [file 12272_2016_816_MOESM1_ESM.doc]
